# MICMIC: identification of DNA methylation of distal regulatory regions with causal effects on tumorigenesis

**DOI:** 10.1186/s13059-018-1442-0

**Published:** 2018-06-05

**Authors:** Yin Tong, Jianlong Sun, Chi Fat Wong, Qingzheng Kang, Beibei Ru, Ching Ngar Wong, April Sheila Chan, Suet Yi Leung, Jiangwen Zhang

**Affiliations:** 1 School of Biological Sciences, The University of Hong Kong, Hong Kong, Hong Kong; 2 Department of Pathology, The University of Hong Kong, Queen Mary Hospital, Pokfulam, Hong Kong

**Keywords:** DNA methylation, Enhancer, Bioinformatics, Cancer, Information theoretic approaches, Epigenetic editing

## Abstract

Aberrant promoter methylation is a common mechanism for tumor suppressor inactivation in cancer. We develop a set of tools to identify genome-wide DNA methylation in distal regions with causal effect on tumorigenesis called MICMIC. Many predictions are directly validated by dCas9-based epigenetic editing to support the accuracy and efficiency of our tool. Oncogenic and lineage-specific transcription factors are shown to aberrantly shape the methylation landscape by modifying tumor-subtype core regulatory circuitry. Notably, the gene regulatory networks orchestrated by enhancer methylation across different cancer types are seen to converge on a common architecture. MICMIC is available on https://github.com/ZhangJlab/MICMIC.

**Electronic supplementary material:**

The online version of this article (10.1186/s13059-018-1442-0) contains supplementary material, which is available to authorized users.

## Background

Appropriate DNA methylation patterns are critical for (epi)genomic stability and gene expression regulation [1]. In particular, it is well established that promoter hypermethylation is a common epigenetic mechanism for tumor suppressor inactivation in cancer [2]. However, many genes lowly expressed in normal samples were not differentially expressed with differentially methylated promoter [3, 4]. Some genes have been verified to be regulated by aberrant promoter methylation with a causal effect on tumorigenesis, including CDKN2B, CDKN2A, RB, APC, BRCA1, and MLH1 [5–7]. Recently, DNA methylation of enhancers in various cancers has been under intense study [4, 8–11]. However, its exact role and whether it is merely a marker of malignancy or a causal factor is largely unknown. Some of these studies focused on well-annotated enhancer regions. However, the annotated enhancer sites are mainly derived from the epigenome profiling of limited cell lines or tissues, lacking an in-depth coverage of distal regulatory sites in patient cancer samples. DNA methylation may be similar to somatic mutations in cancer, in which only a subset of events is causal or “drivers,” while most are “passengers.” To identify the subset that are causal, we need solutions that enable us to: (1) genome-wide identify causal DNA methylation of enhancers and its gene targets in pan-cancers in an unbiased manner; and (2) directly validate a specific methylation event on the putative enhancer by experimentation. Pharmacological inhibition of DNA methylation with the drug 5-azacitidine is commonly used for experimental validation, but it induces genome-wide DNA demethylation without specificity.

In this study, we designed a set of tools for identifying genome-wide DNA methylation of distal regulatory sites that result in a causal effect on tumorigenesis. De novo enhancers/silencers and its direct gene targets were inferred by information theoretic approaches [12, 13] and validated with the emerging CRISPR/dCas9 epigenetic editing [14–17] technique. Information theoretic approaches have been proved effective to distinguish the direct from indirect connection in other applications with solid mathematical proof [18, 19]. Strikingly, we have found that the modulation of DNA methylation on distal regulatory sites by dCas9-DNMT3A-3 L has profound effect on cancer cell behavior similar to promoter methylation, e.g. cell migration and proliferation altered along with target gene expression change, even though the distal regulatory site 200 kb away. By contrast, dCas9-TET1 has the opposite effect on its target gene expression. Our strategy recovered many known enhancers and unannotated regulatory sites from different cancer types, differential methylation of which regulated known or novel tumor-suppressor/oncogene with causal effect on cell malignancy and patient survival. Furthermore, our study also provides mechanistic insight on how DNA methylation of distal regulatory sites is critical for the maintenance of tumor cell identity and malignancy with gene network perspective.

## Results

### Pipeline for MICMIC to infer methylation regulation networks

**Fig. 1:**
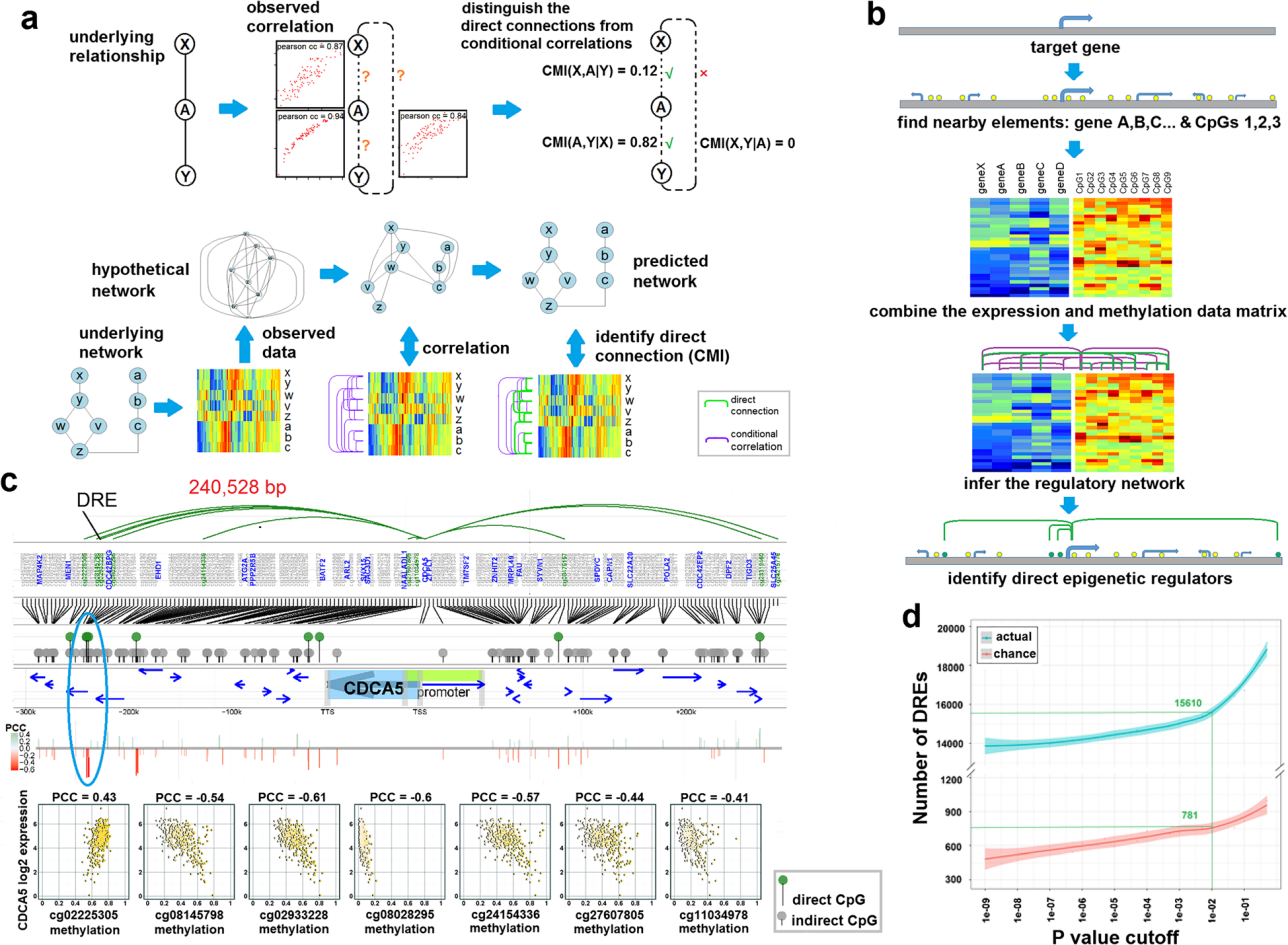
Pipeline for inferring methylation regulation networks. **a** *Top*: *Schematic* of the MICMIC pipeline that uses information theoretic approaches to distinguish direct regulation from indirect correlation, where variables X and Y are connected only via variable A, then CMI(X,Y|A) will be close to zero, suggesting that there is no direct connection between X and Y. *Bottom*: A PC algorithm is used to infer the regulatory network from the observed data matrix, eliminating the indirect edges by CMI testing. **b** MICMIC is designed to identify the regulatory relationship between the methylation level of a CpG probe and the expression level of its potential gene target. For every target gene tested, we included all nearby genes and CpGs ± 300 kb from the transcription start site (TSS) of the test gene and merged the related expression and methylation matrix together. Then MICMIC applies the CMI-based PC algorithm to infer the regulatory network. CpG probes that passed the test were named direct regulatory elements (DREs). The DRE and its gene targets were denoted as DRE-target pairs. **c** A representative example of the MICMIC output for the CDCA5 gene, where ten DREs (nine shown here) were identified to be associated with CDCA5 expression in gastric cancer (TCGA STAD), four of which were at least 240 kb away from the TSS of the target gene. One of these DREs, cg02933228 (*blue oval*), was experimentally verified. In the *lollipop diagram*, *green* represents significant CpG probes, i.e. DREs. The Pearson correlation coefficient (PCC) for each DRE-target pair was represented by a *vertical line* (*red* for negative PCC and *green* for positive PCC). **d** Simulation test to justify the MICMIC *p* value cut-off. The number of actual DREs identified (*blue*) vs the number of DREs identified by chance (*red*) at various *p* value cut-offs

To identify driver methylation events during tumorigenesis, we developed a strategy based on information theoretic approaches to distinguish the direct from indirect correlation between the methylation of CpG probes and the expression of its potential gene targets. Our method, “Methylation Regulation Network Inference by Conditional Mutual Information Based PC-algorithm” (MICMIC), is composed of three layers. The bottom layer uses conditional mutual information (CMI) to determine the dependence relationship between three nodes, genes, and/or CpG probes (Fig. 1a). If variables X and Y are connected only via A, then CMI(X,Y|A) will be close to zero, indicating that there is no direct connection between X and Y. The middle layer uses a path consistency algorithm (PC-algorithm) to infer the regulatory network that includes all nodes (Fig. 1a). To start with, all nodes are considered connected and each edge is tested by CMI based on the observed data. The final network emerges after all false positive connections are eliminated. Finally, in the top layer, MICMIC identifies each CpG probe and its direct target(s) as a pair, denoted here as a DRE-target pair (DRE, direct regulatory elements) (Fig. 1b). Since many methylation events are merely a consequential effect of the cancerous state rather than being causal, MICMIC was purposely designed not to call differentially methylated regions. To identify DRE-target pairs relevant to tumorigenesis, we focused on genes that were determined to be essential for tumorigenesis by differential expression test and master regulator analysis (MRA), which was designed to quantify the enrichment of cancer signature genes among the regulatory neighbors of the target gene (see “Methods”). For each target gene tested, we included all nearby genes and CpG probes ±300 kb away from the transcriptional start site (TSS) of the gene and merged the expression and methylation matrix together. The CMI-based PC algorithm inferred the regulatory network and the DRE-target pair (see “Methods”). We downloaded TCGA level 3 datasets for various cancers, encompassing HumanMethylation450 array and RNA-sequencing (RNA-seq) data. As an example, in the TCGA gastric cancer cohort (STAD) for the gene CDCA5, we identified ten DREs associated with CDCA5 expression, with four of them > 240 kb away from the TSS of CDCA5 (Fig. 1c). Subsequently, we successfully experimentally verified one of these DREs, cg02933228, which will be discussed further below. The false discovery rate (FDR) for MICMIC was 0.05 based on simulation testing (Fig. 1d).

### Genomic features enriched in distal regulatory interactions identified by MICMIC

**Fig. 2:**
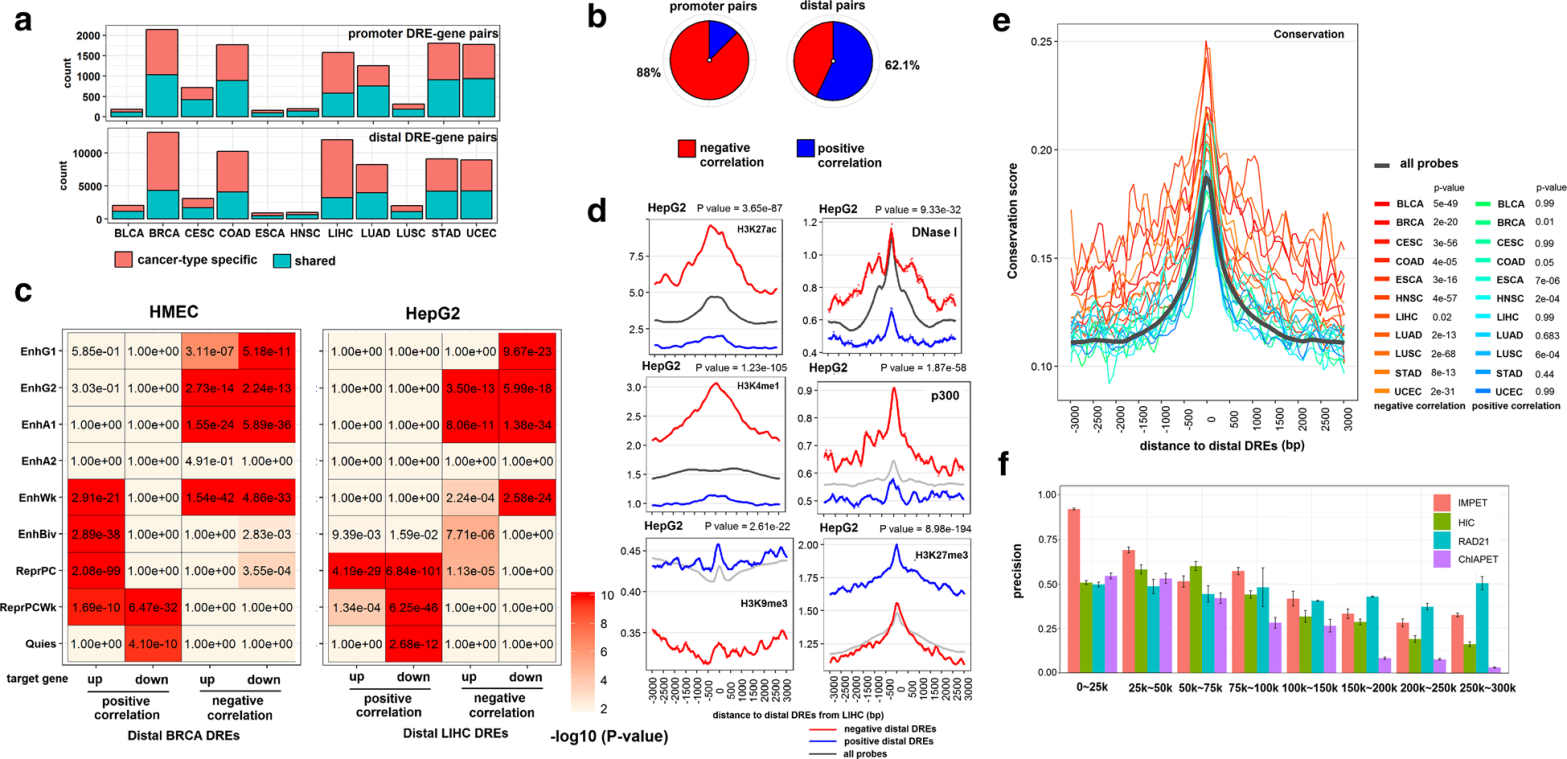
Genomic features of DREs identified across cancers. **a** *Bar chart* showing the number of promoter DRE-gene pairs (*top*) and distal DRE-gene pairs (*bottom*) identified from the TCGA cancer cohorts. *Blue bars* indicate the fraction of the DRE-gene pairs shared by more than one cancer type, while *red bars* indicate the fraction of the cancer-type specific DRE-gene pairs. **b** The negative and positive correlation between DRE methylation and its target gene expression are shown by *red* and *blue*, respectively. Promoter pairs are mainly negatively correlated (88% in total). **c** Representative results showing the preferred chromatin state of distal DREs in liver (HepG2) and breast (HMEC) cancer cell lines. The distal DREs for each cell line were inferred from those identified from the corresponding TCGA cohort, LIHC and BRCA, respectively, here in this example. The number of distal DREs were counted at each chromatin state, with the *heatmap* color and number indicating the enrichment *p* value of distal DREs in each state. Results for other cancer types can be found in Additional file 1: Figure S2. **d** Representative results in liver cancer cell line HepG2, showing increased chromatin signals for H3K27ac, H3K4me1, p300, and DNase I hypersensitivity at genomic regions surrounding the negatively correlated distal DREs, in contrast to increased chromatin signals for repressive marks H3K9me3 and H3K27me3 surrounding the positively correlated distal DREs. *P* values were calculated by t-test comparing the signals of negatively correlated distal DREs vs that of an all probes control. The results for other cancer cell lines can be found in Additional file 1: Figure S4. **e** Average conservation score of distal DRE flanking regions. PhastCons conservation score was in the range of 0–1 (non- to perfectly conserved). *P* values are calculated between distal DREs and all probes (as control) by t-test. **f** The precision of DRE-target pairs predicted by MICMIC was determined by calculating the positive predictive value (PPV) in comparison with other chromatin interaction data, including IM-PET, Hi-C, RAD21-cohesin, and ChIA-PET (see “Methods,” only negatively correlated pairs considered here)

From analysis of 11 different cancer types from the TCGA datasets, the number of DREs was in the range of 2192–13,027 (total 73,255) and the number of DRE-target pairs was in the range of 2234–13,570 (total 80,334). Of DRE-target pairs, 57.4% were cancer specific and 42.6% shared by more than one cancer type. A total of 55,993 DREs that were > 2 kb away from the TSS were termed distal DREs, similar to a previous study [9]. Of the promoter DREs (≤ 2 kb), 88.8% were negatively correlated with their target genes (Fig. 2a, b), among which the majority were downregulated (Additional file 1: Figure S1). The percentage of negative and positive correlations for distal DRE-target pairs were 37.9% and 62.1%, respectively (Fig. 2a, b). To identify enriched genomic features, we used the ENCODE ChromHMM 18-state models to annotate the distal DREs for 6/11 cancer types based on the availability of the corresponding cell line data (see “Methods”) [20]. Of the six tested, all of the distal DREs negatively correlated with its targets were enriched (*p* value < 0.01) in two or more enhancer regions (EnhG1, EnhG2, EnhA1, EnhA2, EnhWk), suggesting that methylation of an enhancer could negatively regulate target gene expression (Fig. 2c and Additional file 1: Figure S2). On the contrary, all of the DREs positively correlated with its targets were enriched in one or two of the repressor regions (ReprPC, ReprRCWk), but not in the enhancer regions (Fig. 2c and Additional file 1: Figure S2). Bivalent Enhancers (EnhBiv), first reported in stem cells [21], were enriched in both negatively and positively correlated DREs. We then compared both negatively and positively correlated DREs for the enrichment of active chromatin marks (H3K27ac, H3K4me1, p300, and DNase I hypersensitivity) and repressive marks (H3K9me3 and H3K27me3). We observed strong enrichment of active marks around the negatively correlated distal DREs and strong enrichment of repressive marks at the positively correlated ones (Fig. 2d and Additional file 1: Figure S4). Enrichment of H3K4me3, marker of active promoters, was only observed at a minority (< 30%) of negatively-correlated DREs, which were 2–3 kb away from TSS (Additional file 1: Figure S4c). Similarly, the PhastCons conservation score reached its peak at the center of the genomic regions flanking negatively correlated distal DREs across all cancer types, and was significantly higher than the control group (*p* value was in the range of 2.18e-68–0.027) (Fig. 2e). By contrast, there is much weaker or no enrichment for positively correlated distal DREs (Fig. 2e). The precision of our distal DRE-target prediction was evaluated by different chromatin interaction datasets, such as IM-PET, Hi-C, RAD21-cohesin, and ChIA-PET [22–24] (see “Methods,” only negatively correlated pairs considered here). The precision rate of MICMIC reached up to 90% when the DRE-target pairs were separated by up to 25 kb and 50% even when the pairs were separated up to 100 kb (Fig. 2f). The TCGA samples analyzed in this study and the DREs identified by MICMIC were listed in the following tables (Additional file 2: Table S1 and Additional file 3: Table S2).

### Validation of causal DNA methylation events involved in tumorigenesis by epigenome engineering techniques in gastric cancer

**Fig. 3:**
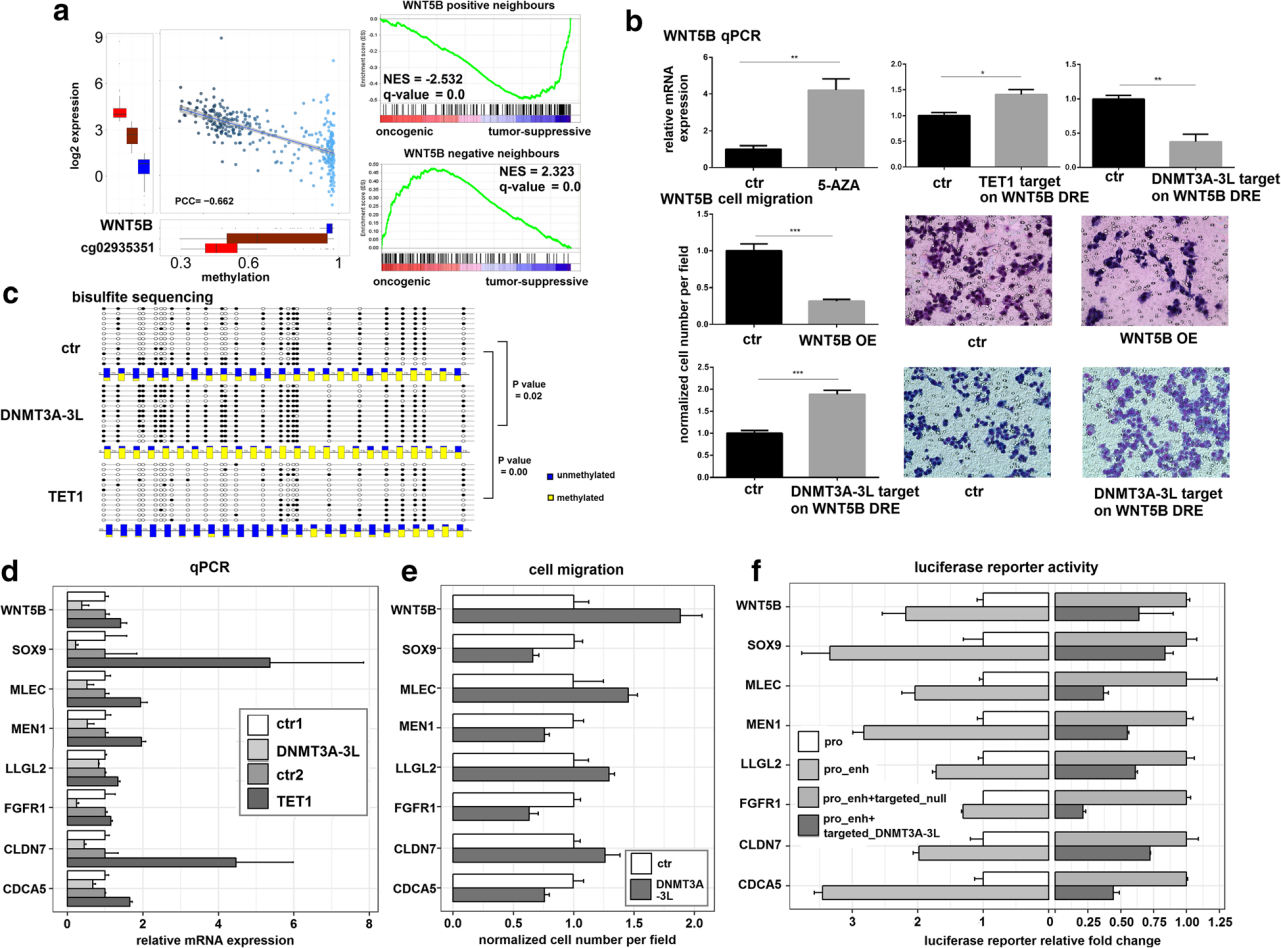
Validation of causal DNA methylation events in gastric cancer. **a** Representative results showing the negative regulation of WNT5B by methylation of its distal DRE (cg02935351, 22,595 bp from WNT5B TSS). *Box plot* shows the high, middle, and low expression groups of WNT5B, plotted against the methylation of the distal DRE in each group. MRA analysis was implemented by the gene set enrichment analysis (GSEA) method. GSEA graphs show tumor-suppressive signatures of WNT5B by MRA. Correlation analysis and MRA for other genes are shown in Additional file 1: Figure S5. **b** Confirmation that methylation of the distal DRE is the causal event for WNT5B regulation and cellular malignancy. qPCR results showing increased WNT5B expression in AGS cells treated with 5-AZA or dCas9-TET1, and decreased expression in cells transfected with dCas9-DNMT3A-3 L relative to controls. Cell migration assays showed that dCas9-DNMT3A-3 L targeting increased cell migration, while overexpression (OE) of WNT5B suppressed tumor cell migration. Significance was determined by t-test and *error bars* represent ± SD. **c** Bisulfite sequencing validation of increased methylation of the CpGs surrounding the dCas9-DNMT3A-3 L targeted DRE of WNT5B. In the *lollipop diagram*, *black circles* stand for methylated Cs and *white circles* for unmethylated Cs. Each *box* below corresponds to one CpG position in the genomic sequence. The *colored bars* summarize the methylation states of all sequences at that position with *yellow* for methylated Cs and blue for unmethylated Cs. **d** qPCR results for eight gastric cancer genes after dCas9-DNMT3A-3 L/TET1 epigenetic editing with dCas9-only as control, labelled as ctr1 and ctr2. Three independent replicates were conducted for each experiment. All DRE-target pairs tested here showed strong anti-correlation between expression and methylation, and the qPCR results showed dCas9-TET1 targeting increased gene expression, while dCas9-DNMT3A-3 L targeting inhibited gene expression (*p* value < 0.01, student t-test). **e** Summary of cell migration assay results for eight gastric cancer genes, showing the causal effects of distal DRE methylation on cancer cell malignancy. See photos in Additional file 1: Figure S9. **f** The distal DRE region and promoter of each gene of interest were cloned into the pGL3 reporter vector and assayed for luciferase activity. The reporter constructs were also co-transfected with dCas9-DNMT3A-3 L (pro_enh + targeted_DNMT3A-3 L), resulting in decreased luciferase activity

**Fig. 4:**
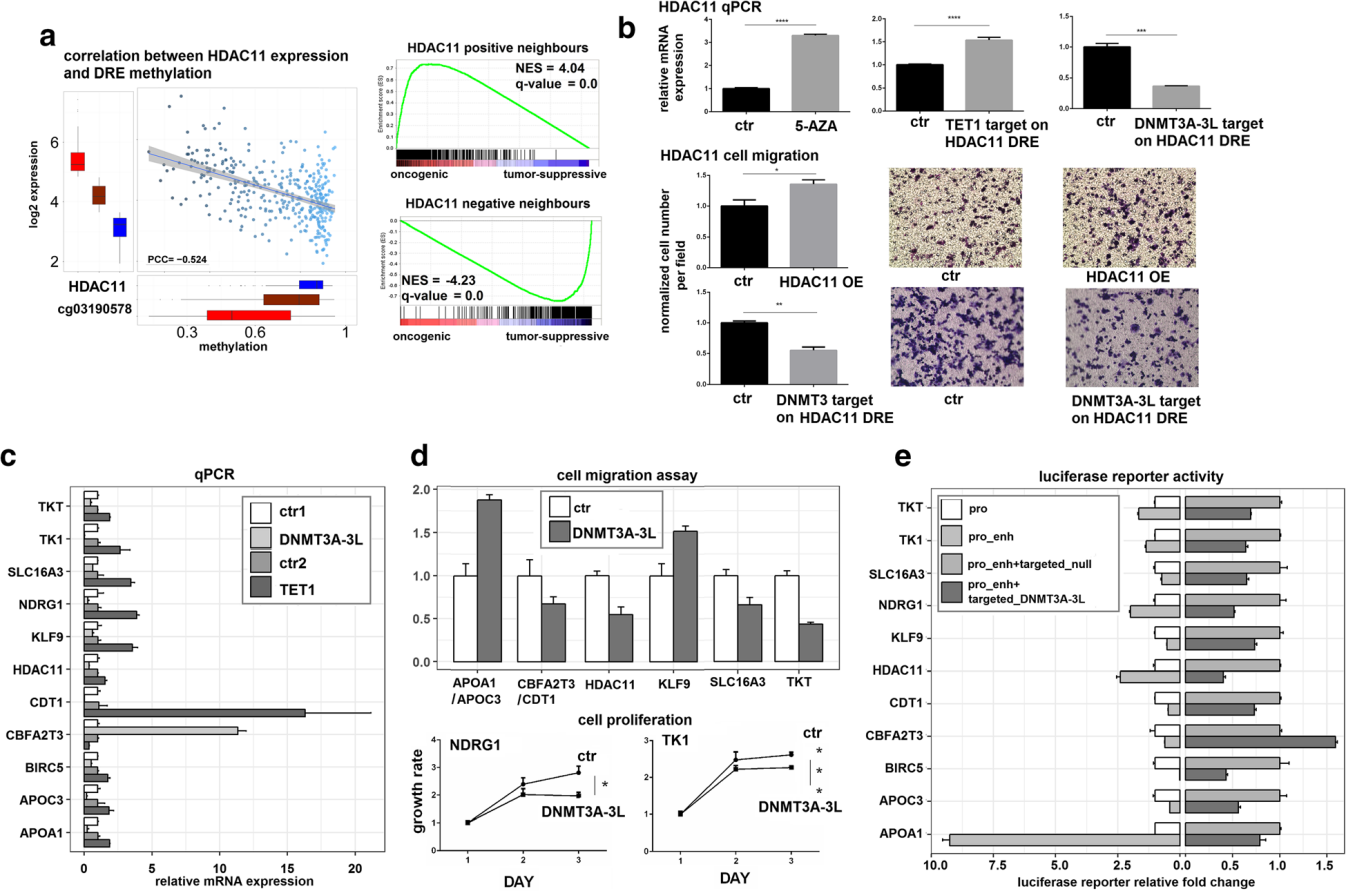
Validation of causal DNA methylation events in liver cancer. **a** Representative results showing the negative regulation of HDAC11 by methylation of its distal DRE (cg03190578, 3817 bp from HDAC11 TSS). *Box plot* shows the high, middle, and low expression groups of HDAC11, plotted against the methylation of the distal DRE in each group. *GSEA graphs* show oncogenic signatures of HDAC11 by MRA. Correlation analysis and MRA for other genes are shown in Additional file 1: Figure S10. **b** Confirmation that methylation of the distal DRE is the causal event for HDAC11 regulation and cellular malignancy. qPCR results showing increased HDAC11 expression in PLC8024 cells treated with 5-AZA or dCas9-TET1, and decreased expression in cells transfected with dCas9-DNMT3A-3 L relative to controls. Cell migration assays showed that dCas9-DNMT3A-3 L targeting suppressed cell migration, while overexpression (OE) of HDAC11 increased tumor cell migration. Significance was determined by t-test and *error bars* represent ± SD. **c** qPCR results for 11 liver cancer genes after dCas9-DNMT3A-3 L/TET1 epigenetic editing with dCas9-only as control, labelled as ctr1 and ctr2. Three independent replicates were conducted for each experiment. Ten out of 11 DRE-target pairs were predicted to be negatively regulated by DRE methylation, CBFA2T3 was predicted to be positively regulated by methylation of the distal DRE (cg20283771).The qPCR results (*p* value < 0.01 by Student’s t-test) were consistent with the predictions (Additional file 1: Figure S10). **d** Summary of results for cell migration and proliferation assays for liver cancer genes, showing the causal effects of distal DRE methylation on cancer cell malignancy. See photos in Additional file 1: Figure S11. **e** The distal DRE region and promoter of each gene of interest were cloned into the pGL3 reporter vector and assayed for luciferase activity. The reporter constructs were also co-transfected with dCas9-DNMT3A-3 L (pro_enh + targeted_DNMT3A-3 L), resulting in decreased luciferase activity except CBFA2T3 positively correlated with its DRE methylation

We chose distal DRE-target pairs for validation if: (1) there was a strong correlation between expression and methylation, represented by a significant Pearson correlation coefficient (PCC < − 0.3 or > 0.3); and (2) the target gene was determined to be essential for tumorigenesis by differential expression test and MRA (see “Methods,” Figs. 3a and 4a, and Additional file 1: Figures S5 and S7). For example, in gastric cancer, WNT5B expression and methylation of its distal DRE (cg02935351) were strongly anti-correlated and WNT5B was predicted to be a tumor suppressor by MRA (Fig. 3a). Next, we performed epigenetic editing by using CRISPR-dCas9 based technologies, such as the casilio system [14] for targeted methylation with a DNMT3A-3 L fusion protein and the dCas9-SunTag scaffold with scFv–TET1 catalytic domain fusions [15] for targeted demethylation to the intended genomic sites in the AGS human gastric cancer cell line (Additional file 4: Table S3). Remarkably, targeting DNMT3A-3 L to the region near cg02935351 downregulated WNT5B, while targeting TET1 to this region produced similar results to treatment with the global DNA methylation inhibitor, 5-AZA, and upregulated WNT5B (Fig. 3b). The effect of targeting DNMT3A-3 L/TET1 to the distal DRE site of WNT5B was confirmed by bisulfite sequencing without off-target on other genes (Fig. 3c and Additional file 1: Figure S7a). We then tested if modulation of the DNA methylation of distal DREs could affect cell migration. Strikingly, cancer cell migration increased as a result of DNMT3A-3 L targeting, but decreased by TET1 targeting or overexpression of WNT5B complementary DNA (cDNA) (Fig. 3b and Additional file 1: Figure S9). To further confirm the regulatory function of this distal DRE region, we cloned a 1-kb genomic region flanking cg02935351 and the WNT5B promoter into the pGL3 luciferase reporter vector and verified its putative enhancer status (Fig. 3f). Interestingly, co-transfection with dCas9-DNMT3A-3 L was also able to regulate the reporter constructs (Fig. 3f). In addition, we verified several other genes, including MLEC, LLGL2, CDCA5, MEN1, CLDN7, SOX9, and FGFR1 by epigenetic modulation of distal DREs followed by quantitative polymerase chain reaction (qPCR), migration assay, and luciferase reporter assay (Fig. 3d–f, Additional file 1: Figure S9). We performed experiments using scrambled single guide RNA (sgRNA), “untargeted,” or catalytically inactive DNMT3A-3 L/TET1 to rule out the possibility of off-target due to overexpression DNMT3A-3 L/TET1 (see “Methods” and Additional file 1: Figure S6). Overall, our experimental results were fully consistent with MICMIC predictions. As mentioned above in Fig. 1, MICMIC predicted a distal DRE for CDCA5, cg02933228, which is > 240 kb away from the TSS of CDCA5 (Fig. 1c), but we were able to achieve robust regulation of this distal DRE with dCas9 epigenetic editing (Fig. 3d–f). This same distal DRE, cg02933228, was also predicted to control the gene MEN1, which we were also able to experimentally confirm. Additionally, our study is the first to show evidence of the gene Malectin (MLEC) being a tumor suppressor (Fig. 3d and e, Additional file 1: Figures S5 and S9). Taken together, MICMIC along with MRA was able to identify causal events in tumorigenesis involving DNA methylation of distal regulatory regions, which we were able to verify via epigenetic editing by dCas9 fused with TET1 or DNMT3A-3 L and identify novel oncogenes/tumor-suppressors in the process.

### Validation of causal DNA methylation events involved in tumorigenesis by epigenome engineering techniques in liver cancer

We also validated MICMIC predictions in liver cancer. First, we observed a strong anti-correlation between HDAC11 expression and cg03190578 methylation (Fig. 4a). As expected, targeted methylation with DNMT3A-3 L to the cg03190578 region decreased HDAC11 expression, while targeted demethylation with TET1 dramatically increased HDAC11 expression (Fig. 4b). Consequently, we found that modulation of DNA methylation on the distal DRE, cg03190578, by dcas9-DNMT3A-3 L significantly decreased cancer cell migration suggesting an oncogenic function for HDAC11 in liver cancer, which was confirmed by increased cell migration upon overexpression of HDAC11 (Fig. 4b). In contrast, dcas9-TET1 targeting to the cg03190578 region increased cancer cell migration (Additional file 1: Figure S11). In addition to HDAC11, we validated other genes as well and identified the distal DREs of HDAC11, APOA1, NDRG1, TK1, and TKT to be enhancers and the distal DREs of BIRC5, CDT1, CBFA2T3, SLC16A3, KLF9, and APOC3 to be silencers (Fig. 4c–e, Additional file 1: Figures S10 and S11). Among these genes, some shared the same distal DRE, e.g. APOA1 shared cg23193059 with APOC3 and CDT1 shared cg20283771 with CBFA2T3. Intriguingly, methylation of DRE cg20283771 was positively correlated with CBFA2T3 expression, but negatively correlated with CDT1 (Fig. 4c, Additional file 1: Figure S10). Both genes were verified to be causally regulated by methylation of cg20283771 with combined effect on cancer cell migration after dCas9-DNMT3A-3 L targeting (Fig. 4c–e). For two genes, NDRG1 and TK1, there was no significant difference in cell migration after dCas9-DNMT3A-3 L targeting of their distal DREs, but they did show a significant decrease in cell proliferation (Fig. 4d).

### Aberrant methylation landscape of distal DREs can be shaped by oncogenic and lineage-specific transcription factors (TFs) with profound effects on tumorigenesis and patient survival

**Fig. 5:**
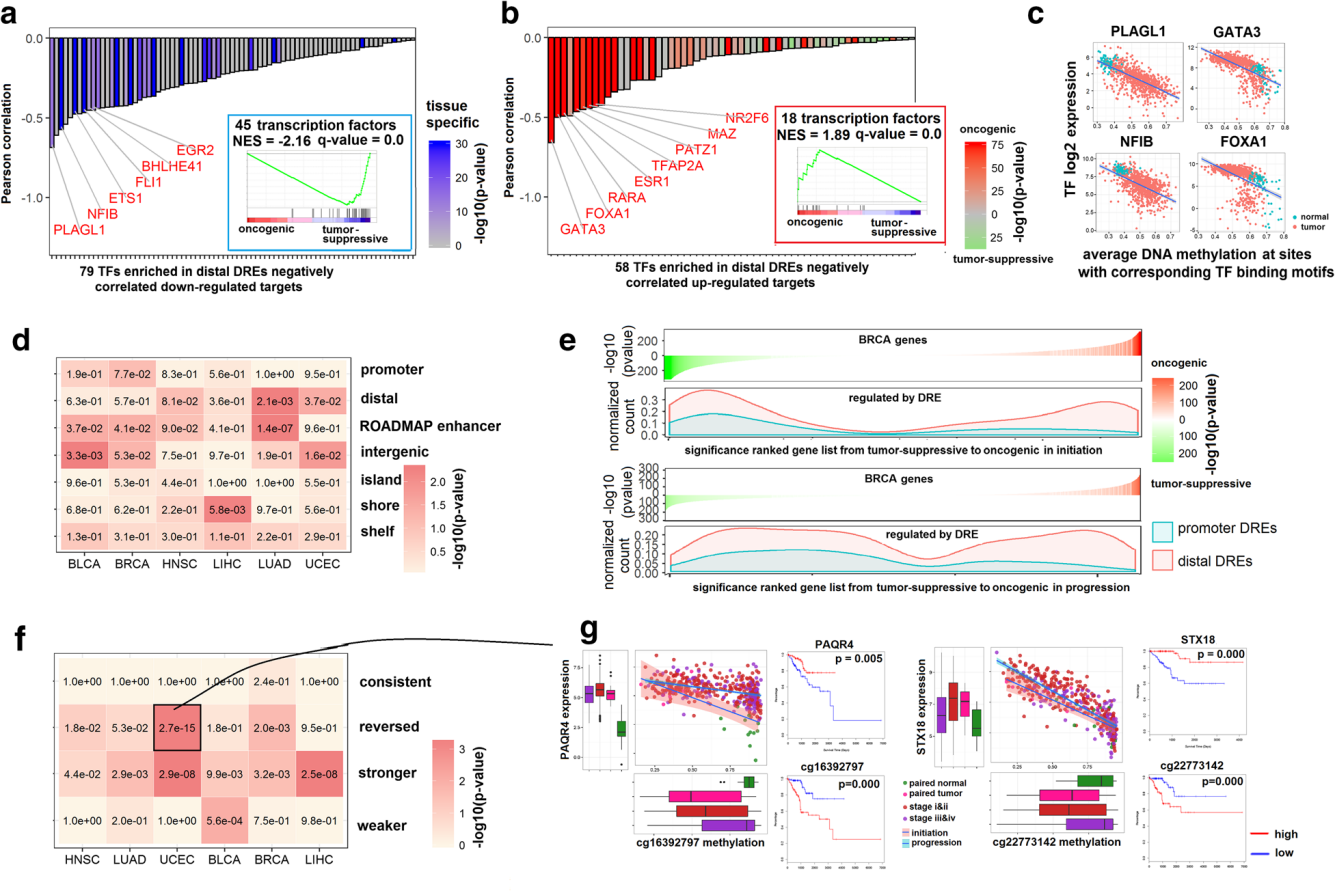
Interplay between distal DRE methylation and the cognate transcription factor binding has profound effects on tumorigenesis. *Bar charts* show the enriched transcription factors (TFs) that bind to DREs showing a negative correlation between the average methylation of TF binding motifs and TF expression, with (**a**) downregulation (negative-down DREs) or (**b**) upregulation (negative-up DREs) of the target gene. Intensity of *blue color* indicates the degree of tissue specificity of the TF in breast cancer (BRCA) compared to other tissue types. Intensity of *red*/*green color* indicates the degree of oncogenic/tumor suppressive behavior of the TF. *Bar chart* showing the enriched TFs binding on the group-specific distal DREs. The TFs were ranked by the negative correlation between the TF expression and average DNA methylation of the TF binding motifs on the group-specific distal DREs. The correlation value is shown on the *y-axis*. *Colors* represent the tissue type significance or master regulator significance of the TF gene. The cancer signature association of the TFs is shown in the inset *GSEA plot*. **c** Representative examples of TFs that showed a negative correlation between the expression of the TF and average DNA methylation of the TF binding motifs on distal DREs. **d** *Heatmap* showing the enrichment significance of DREs associated with patient survival in various genomic regions. The *heatmap* color and number indicates the enrichment *p* value of DREs associated with patient survival in each category. **e** Higher impact of distal DREs on cancer genes compared with promoter DREs during initiation (*top*) and progression (*bottom*). *Y-axis* for the two top *waterfall plots* indicates the master regulator significance for each gene, ranked from tumor-suppressive to oncogenic. *Y-axis* for the two *bottom panels* shows the density of normalized gene counts controlled by promoter or distal DREs. See results of other cancers in Additional file 1: Figure S16. **f** *Heatmap* showing the enrichment significance of distal DREs associated with patient survival in the four methylation patterns of distal DREs, i.e. “consistent,” “reversed,” “stronger,” and “weaker” according to the direction of methylation change from the initiation to progression stage of tumorigenesis for each cancer type. The number in each square represents the *p* values. **g** Example of two distal-DRE target pairs identified in uterine cancer that show a “reversed” methylation pattern. *Box plots* on the *x-axis* show the DRE is demethylated during the initiation stage but becomes remethylated during cancer progression. High methylation of both DREs and low expression of their target genes were associated with poorer patient survival

We next investigated how TFs can regulate and shape the methylation landscape of distal DREs in cancers (see “Methods”). First, we categorized all distal DREs in each cancer into four subgroups, i.e. negative-up, negative-down, positive-up, and positive-down, dependent on whether the pair was negatively or positively correlated and whether the target gene was up- or downregulated in tumor versus normal samples. After identification of TFs associated with distal DREs (Additional file 1: Figure S12), we calculated the PCC between the expression level of each enriched TF and the average methylation level of its cognate binding sites on distal DREs for each subgroup (Fig. 5c) and ranked TFs by its PCC in ascending order. Strikingly, the top ranked TFs identified from the negative-down group were mostly tissue-specific TFs across various cancer types, whereas TFs identified from the negative-up group were mainly oncogenic TFs (Fig. 5a and b, Additional file 1: Figure S13). GSEA further confirmed that these tissue-specific TFs are tumor suppressors (Fig. 5a inset), suggesting that hypermethylated distal DREs from the negative-down group in conjunction with the decreased expression of the cognate tissue-specific TFs, lead to downregulation of its distal gene targets in cancer. Similarly, GSEA confirmed that the top ranked TFs in the negative-up group were enriched for oncogenic TFs (Fig. 5b inset and Additional file 1: Figure S13), suggesting that hypomethylation of distal DREs from negative-up group together with the increased expression of the cognate oncogenic TFs, consequentially lead to upregulation of its distal gene targets. For distal DREs positively correlated with its targets, we found significant enrichment of TFs with repressor activity (*p* value = 8e-7), suggesting that DNA methylation may affect the binding of TF repressors with implications in tumorigenesis (Additional file 1: Figure S14).

We also investigated the association between DRE methylation and patient survival. We identified 1081 DRE methylation correlated with patient survival (q-value < 0.1, FDR by BH procedure) in bladder cancer (BLCA), breast cancer (BRCA), head and neck carcinoma (HNSC), liver cancer (LIHC), lung cancer (LUAD), and uterine corpus endometrial cancer (UCEC). For BLCA, the DREs associated with survival were enriched in intergenic regions. For LUAD and UCEC, the DREs associated with survival were enriched in distal regions (enrichment *p* value < 0.05) (Fig. 5d). We then calculated the number of master cancer genes (via MRA) that are regulated by DNA methylation of the promoter or distal DREs and used the density distribution to quantify the effect that methylation of those DREs have on tumorigenesis (Fig. 5e, Additional file 1: Figures S15 and S16). The results indicated that the methylation of distal DREs compared to proximal DREs had more of an impact on the regulation of both oncogenes and tumor suppressors at the initiation and progression stage of tumorigenesis.

Furthermore, we analyzed the dynamic change in methylation patterns that can occur at distal DREs as tumors transition from the initiation to the progression stage. During this transition, methylation patterns of distal DREs can remain the same (“consistent”), become differentially methylated in the opposite direction (“reversed”), or show increased (“stronger”) or decreased (“weaker”) methylation change in the initiation versus the progression stage (Additional file 5: Table S4). Strikingly, distal DREs related to patient survival were more enriched in the “reversed” group (Fig. 5f). For example, in uterine cancer, the distal DRE of PAQR4 was de-methylated at the initiation stage but became re-methylated in higher stage tumors. Moreover, the high methylation of the DRE and lower expression of PAQR4 were correlated with poorer patient survival (Fig. 5g). Many more distal DRE-target pairs fell into this category, including the gene STX18 (Fig. 5g).

### Diverged tumor-subtype core regulatory circuitry and converged pan-cancer global topology of TF network associated with distal DRE

**Fig. 6:**
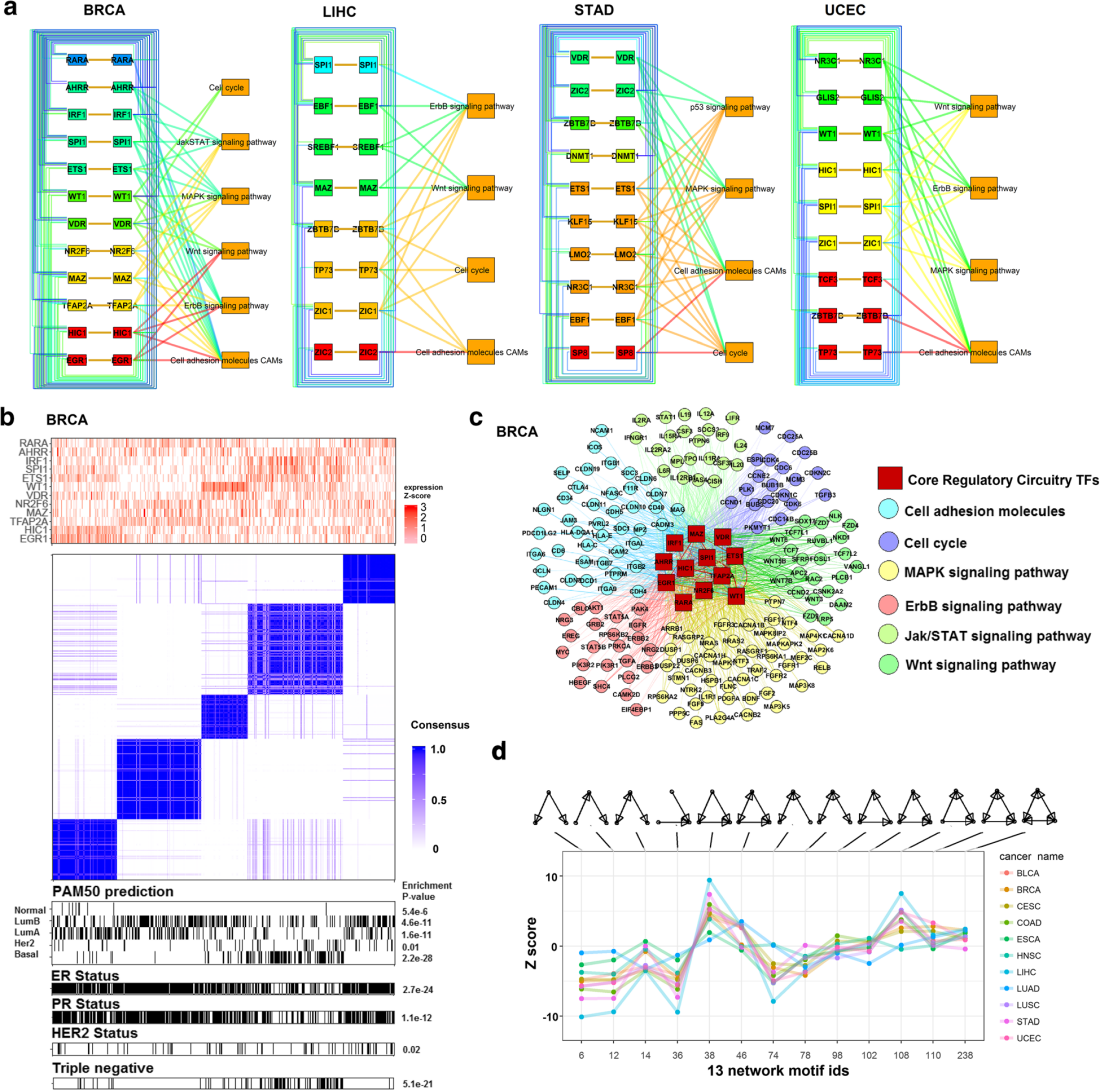
Tumor-subtype core regulatory circuitry and pan-cancer global topology of TF network regulated by DNA methylation of distal DREs. **a** The interconnected auto- and cross-regulation loops within the CRC TFs. The links between TFs were derived from distal DRE-target pairs in which the DRE harbors binding sites for the CRC TFs. The TFs are colored by the tumor subtypes in which they are highly expressed. Effects of the CRC on tumorigenesis are analyzed by the cancer pathway enrichment of the TFs’ targets (hypergeometric *p* value < 0.05), representing in the right side of the CRC. **b** *Top*: *Heatmap* of the expression Z-score of CRC TFs in the tumor subtypes. *Bottom*: Joint consensus clustering by the expression of CRC TFs and methylation of binding DREs shows a great similarity between the CRC subtypes and PAM50 subtypes in breast cancer. See results of other cancers in Additional file 1: Figures S17–S19. **c** Signaling pathways in breast cancer regulated by CRC TFs whose targets were identified by the distal DRE-target pairs in which the DRE harbored the TF binding sites. Each *color* of a gene node indicates a different cancer pathway. *Edges* represent regulatory relationships. **d** Convergence of network topology across cancer types (see “Methods”). For each cancer type, their TF networks were decomposed and categorized into 13 different types of basic three-node network motifs, indicated by the topology structures above the graph. The *X-axis* shows the numerical identification number associated with each motif. The relative enrichment (Z > 2) or depletion (Z < − 2) of each of the 13 basic network motifs for each cancer type was calculated as a Z-score (*Y-axis*)

Multiple lines of evidence have indicated that super-enhancers (SEs) with associated oncogenic TFs play a pivotal role in regulating and maintaining tumor cellular identity [25, 26]. It has been shown that SEs function as a platform to integrate a set of key TFs forming a core regulatory circuitry (CRC) to regulate tumor-subtype specific gene expression. The TFs in each CRC are auto-regulated by itself through binding sites on its corresponding SE. The TFs can also cross-regulate each other by forming an interconnected loop with cognate binding sites on other TFs’ related SEs. Based on this information, we took advantage of the genome-wide information of distal DRE-target derived from MICMIC to assemble the CRCs regulated by DNA methylation for each cancer type (Additional file 6: Table S5). We hypothesized that cancer subtypes could be distinguished by the joint consensus clustering of the DNA methylation of each TF’s cognate binding site and the expression level of the corresponding TF. Strikingly, the joint consensus clustering with the assembled CRCs for cancers, including breast, liver, stomach, and endometrial carcinoma, can identify the cancer subtypes in line with the previously established molecular/pathological subtypes. For instance, breast cancer subtypes (lumA, lumB, and basal like) [27] can be identified by the joint clustering (Fig. 6b). We can further identify the underlying signaling pathways regulated by CRC in different subgroups (Fig. 6c and Additional file 1: Figures S17–S19). Similarly, the global gene regulatory network (GRN) for each cancer can be generated with the information of our genome-wide distal DRE-target interaction and TFs associated with each DRE. Topology of GRN can be compared based on the normalized frequency of the three-node network motif in each cancer [28, 29]. Notably, GRNs across various cancer types converged on a common architecture (Additional file 7: Table S6), highlighting the similarity of GRN controlled by DNA methylation of distal regulatory regions at the higher-order organization level (Fig. 6d).

## Discussion

In this study, we aimed to identify DNA methylation of distal regulatory regions with causal effects on tumorigenesis. MICMIC is different from other currently available methylation analysis software in two respects. First, since many methylation events are merely a consequence of epigenetic disruption and not the cause, rather than calling differentially methylated regions first, we begin by: (1) using genes essential for tumorigenesis by differential expression test and MRA to find its distal DRE(s); and (2) take novel application of information theoretic approaches in DRE-target call. Interestingly, about 23.7% putative enhancers flanking our distal DREs harbor known COSMIC non-coding mutations in liver cancer (Additional file 8: Table S7). This can help prioritize the somatic mutations locating on distal regulatory sites as cancer risk loci non-coding variants are enriched in enhancers [25, 30].

Our bench validation with dCas9 targeting is dependent on the experiment with co-transfection of multiple plasmids into cancer cell lines that have to be effectively transfected. This could be challenging for certain cancer types, e.g. only one gastric cell line “AGS” (over 50% transfection efficiency with lipofectamine3000) and a few liver cancer lines have acceptable transfection efficiency in our hand. However, the DNA methylation level seems quite heterogeneous for most DREs in the same cell line. For instance, we can increase or decrease the DNA methylation level of the same DRE site in AGS cell line by dCas9 targeting, and consequentially change the gene expression level in both directions, upregulation or downregulation.

It is common for a single enhancer to control more than one gene and vice versa. As shown above, both oncogene CDCA5 and tumor-suppressor MEN1 were verified to be regulated by the same distal DRE cg02933228. However, the decreased cell migration phenotype after dCas9-DNMT3A-3 L targeting of cg02933228 was only consistent with CDCA5’s function prediction. We need to take into account this complexity when interpreting the phenotypic output from the methylation modulation by dCas9 targeting since the output could be the combined effect of multiple genes targeted by the same distal DRE.

Our study provides mechanistic insight on how DNA methylation of distal DREs is critical for the maintenance of tumor cell identity and malignancy. We found that oncogenic and lineage-specific TFs shape the methylation landscape of distal DREs, which is controlled in concert by the expression level of each enriched TF and the average methylation level of its cognate binding sites on distal DREs. Key TFs were identified to be part of core regulatory circuitries (CRCs) associated with distal DREs for regulation of tumor-subtype specific gene expression. Furthermore, we showed that the network topology of GRN derived from DNA methylation of distal DREs may have the same architecture across different cancer types, enriched for network motifs like “feed forwards loop,” “regulated mutual,” and “regulating mutual.” This similarity in topology suggests that a common organization principle governs this type of biological networks regulated by DNA methylation of distal regulation regions.

## Conclusions

In this study, we have developed a set of tools to genome-wide identify DNA methylation in distal regions with causal effect on tumorigenesis. Novel oncogenes/tumor-suppressors and their putative enhancers can be identified together based on this strategy. We have extensively validated many of the predictions by epigenetic editing. Our study reveals the prevalent regulation of genome-wide putative enhancers by DNA-methylation with causal effect on cellular malignancy and patient survival. Our study also provides mechanistic insight on how DNA methylation of distal regulatory regions is critical for the maintenance of tumor cell identity and malignancy.

## Methods

### Data collection

We downloaded TCGA level 3 DNA methylation data, clinical data, and RNA-seq data for 4747 matched samples encompassing 11 cancer types: bladder urothelial carcinoma (BLCA); breast invasive carcinoma (BRCA); cervical squamous cell carcinoma and endocervical adenocarcinoma (CESC); colon adenocarcinoma (COAD); esophageal carcinoma (ESCA); head and neck squamous cell carcinoma (HNSC); liver hepatocellular carcinoma (LIHC); lung adenocarcinoma (LUAD); lung squamous cell carcinoma (LUSC); stomach adenocarcinoma (STAD); and uterine corpus endometrial carcinoma (UCEC) (Additional file 2: Table S1). The methylation data is based on the Infinium HumanMethylation450 BeadArray platform, in which the probes covered 485,000 CpG sites across the genome.

### Mutual information and conditional mutual information

Mutual information (MI) is a general measurement of dependence between individual events. This method is based on the joint probability of events to infer dependence without making any assumptions about the nature of their underlying relationships. MI is based on information theory and can be calculated by the entropy of variables. For any variable A, the entropy H(A) is the average amount of information gained from a measurement. And it can be defined by:

1 $$ H(A)=-\sum \limits_{i=1}^{N_A}p\left({a}_i\right)\log p\left({a}_i\right) $$

where p(a) is the probability of any possible value of A. The joint entropy of two discrete systems A and B is defined by

2 $$ H\left(A,B\right)=-\sum \limits_{i=1}^{N_A}\sum \limits_{j=1}^{N_B}p\left({a}_i,{b}_j\right)\log p\left({a}_i,{b}_j\right) $$

where the p(a,b) is the joint probability. When both A and B are independent events, the joint entropy of A and B can be denoted by:

3 $$ H\left(A,B\right)=H(A)+H(B) $$

For any dependent events A and B, the joint entropy will follow:

4 $$ H\left(A,B\right)<H(A)+H(B) $$

The mutual information of I(A,B), which quantifies the dependence of A and B, is defined as the difference between H(A) + H(B) and H(A,B):

5 $$ I\left(A,B\right)=H(A)+H(B)-H\left(A,B\right) $$

6 $$ I\left(A,B\right)=\sum \limits_{i=1}^{N_a}\sum \limits_{j=1}^{N_b}p\left({a}_i,{b}_j\right)\frac{p\left({a}_i,{b}_j\right)}{p\left({a}_i\right)\ast p\left({b}_j\right)} $$

A higher MI represents a greater connection between the events.

To further study the dependence within three or more variables, conditional mutual information (CMI) is introduced to assess the exclusive dependence between any pairs of variables given the value of a third one. CMI can distinguish pairs directly from indirectly connected. The conditional mutual information (CMI) can be calculated by:

7 $$ I\left(X;Y|Z\right)=\sum \limits_{z\in Z}\sum \limits_{y\in Y}\sum \limits_{x\in X}{p}_{X,Y,Z}\left(x,y,z\right)\log \frac{p_Z(z){p}_{X,Y,Z}\left(x,y,z\right)}{p_{X,Z}\left(x,z\right){p}_{Y,Z}\left(y,z\right)} $$

or in terms of entropy:

8 $$ I\left(X;Y|Z\right)=H\left(X,Z\right)+H\left(Y,Z\right)-H\left(X,Y,Z\right)-H(Z) $$

where p(X,Y,Z) is the joint probabilities and H(X,Y,Z) is the joint entropy. A high value for CMI(X,Y|Z) would mean X and Y are directly connected and do not rely on the given variable Z.

We used the kernel density estimation (KDE) to estimate the probability distribution of continuous variables, such as gene expression. KDE was found to be superior to the histograms estimator and the estimation of probability distribution by KDE has been used in MI calculation as follows [18, 19],

$$ P\left({X}_i\right)=\frac{1}{N}\sum \limits_{j=1}^N\frac{1}{{\left(2\pi \right)}^{n/2}{\left|C\right|}^{n/2}}\exp \left(-\frac{1}{2}{\left({X}_j-{X}_i\right)}^T{C}^{-1}\left({X}_j-{X}_i\right)\right) $$

(9)

where C is the covariance matrix of X and |C| is the determinant of matrix C.

From Eqs. 1, 6, and 9, we got the entropy of variable X, MI of (X,Y), and CMI of (X,Y|Z) as:

10 $$ H(X)=\log \left[{\left(2\pi e\right)}^{\frac{n}{2}}{\left|C\right|}^{1/2}\right], $$

11 $$ I\left(X,Y\right)=\frac{1}{2}\log \frac{\mid C(X)\mid \bullet \mid C(Y)\mid }{\mid C\left(X,Y\right)\mid }, $$

12 $$ I\left(X,Y|Z\right)=\frac{1}{2}\log \frac{\mid C\left(X,Z\right)\mid \bullet \mid C\left(Y,Z\right)\mid }{\mid C(Z)\mid \bullet \mid C\left(X,Y,Z\right)\mid }. $$

MI and CMI were normalized by:

13 $$ \widehat{I}\left(X,Y\right)=\frac{I\left(X,Y\right)}{\max \left(I\left(X,Y\right)\right)}, $$

14 $$ \widehat{I}\left(X,Y|Z\right)=\frac{I\left(X,Y|Z\right)}{\max \left(I\left(X,Y|Z\right)\right)}, $$

where maximal(MI) and maximal(CMI) were the MI and CMI values when Y was totally dependent on X. Then, the normalized MI and CMI value were between 0 and 1.

### Significance level determination

To determine the significance level of our MI and CMI examination, we used random permutation and Fisher’s Z statistics to calculate the z-score and *p* value [19]. We randomly shuffled the vectors X and Y many times and got the correlation r between random X,Y (CMI). Then we transformed r to z by:

15 $$ {\mathrm{z}}^{'}=.5\left[\ln \left(1+\mathrm{r}\right)-\ln \left(1-\mathrm{r}\right)\right] $$

The confidence interval would be:

16 $$ {z}^{\hbox{'}}\pm z{\sigma}_{z\hbox{'}} $$

Here, the σ_z_ is the standard deviation of z. We used the observed X,Y value to get the observed CMI value and transformed it into Z-value. The Z-score was calculated by Z score = (Z value-z’)/σ_z_. And the *p* value was calculated by 2*pnorm(−|Zscore|).

### Differential expression analysis

We used the Voom method to normalize the RNA-seq data and calculated the gene differentially expressed between tumor and normal by limma package [31]. We selected the differentially expressed genes (DEGs) by the cut-off of |log2FC| > 0.58 (i.e. fold change cut-off either upregulation > 1.5-fold or downregulation at least 1.5-fold) and adjusted *P* < 0.01, and the DEG list was used for downstream analysis, e.g. identification of the corresponding DREs and master regulator analysis.

### Master regulators analysis (MRA)

Master regulators (MRs) control a large number of downstream targets that play important roles in cancer stage transition. Here, we exploited a classical strategy to identify MRs for cancer initiation (paired tumor vs normal samples) and progression (late-stage [IV] vs early stage [I] samples). The basic framework contains two parts: (1) based on cancer specific gene expression profile, transcriptional targets (termed as regulon) of TFs are inferred using ARACNe [32] with default parameters. Data processing inequality (DPI) was set to reduce the number of indirect connections; (2) gene set enrichment analysis with R gage package [33] is conducted to evaluate whether the regulon of TFs is enriched in the signature of cancer-related phenotype transition (ranked gene list using t value from differential expression analysis). Specifically, the regulon genes of a TF are divided into positive (+) and negative (−) groups based on the Spearman’s correlation coefficients between the expression level of the TF and each gene in its regulon. Then, two runs of gene set enrichment analysis are carried out to determine the MR is activated (i.e. oncogenic) or repressed (i.e. tumor-suppressor): run 1 regulon (+) in from the upregulated side and regulon (−) from the downregulated side; run 2 regulon (+) in from the downregulated side and regulon (−) from the upregulated side. In each run, the enrichment q-values are calculated by Fisher’s method. Regulon(+) of a gene is also called positive neighbors and regulon(−) of a gene is called negative neighbors in this paper. Whichever of the two runs gives the more significant q-value is used as the final q-value; the MR is predicted as oncogenic (the q value in run 1 < the q value in run 2) or tumor-suppressive (the q-value in run 1 > the q-value in run 2) correspondingly.

### Identification of the direct regulatory elements by MI/CMI based PC-algorithm

For genes being tested, we identified the DREs by the following steps:

1. Data preparation. We selected neighboring elements (i.e. messenger RNA expression and CpG probe methylation) of a target gene within a genomic range (default ± 300 kb from TSS of the gene) and integrated the data value for these selected elements (e.g. expression and methylation value). The final result was a data matrix in which columns correspond to samples and rows to variables (i.e. gene or CpG probes). We chose genomic range ± 300 kb since it was reported that the enhancer-promoter interactions peak around 120 kb upstream of the TSS [34].
2. Identification of DREs for the gene on test. We used the network inference method called PC algorithm to infer the regulatory network based on the MI/CMI connections [12]. The PC algorithm is computationally feasible and very efficient for sparse connections frequently encountered in biological networks. The result returned an adjacent matrix representing the direct connected edges. First, we assumed all nodes connected by default to generate a completely connected graph between all genes and all CpG probes within the genomic range (default ± 300 kb from TSS of the gene). Second, MI was calculated for any node pair, e.g. node i and j based on their values in samples. Third, the edge between i and j will be kept in the network only if their MI passes the significance testing (cut-off *p* < 0.01). Fourth, all of the common partners (k) for the i and j pair surviving last test will be used to calculate the CMI(i,j|k), which can distinguish if i-j connection is conditional on variable k. Fifth, we generated a directly connected network in an adjacent matrix after deletion of these indirectly connected edges. Herein, a mutual information cutoff (MI > 0.1 bits) was used to remove weak connections. Finally, we generated a list of the DRE-target pairs that were directly connected.
3. Classification of the DRE-target pairs. The DREs were classified based on the target gene expression (up- or downregulation in tumors), direction of correlation with its target gene expression (positive or negative), and the distance from the TSS of its target gene. DREs locating within ± 2000 bp of the TSS of its target genes were classified as the promoter DREs and others were classified as distal DREs.

The MICMIC pipeline can be adjusted to handle genomic range beyond ± 300 kb. We chose genomic range ± 300 kb here since it was reported that the enhancer-promoter interactions peak around 120 kb upstream of the TSS [34].

Examples of the indirectly correlated CpG-gene pairs rejected by our method are presented in Additional file 1: Figure S3c. The deregulations of the CIMP genes are controlled by the hypermethylation of genome-wide CpG islands and the strongly correlated CpGs rejected by our method showed no correlation in the non-CIMP samples.

### Mapping chromatin state of DREs by ChromHMM 18-state model

To annotate the DREs, we downloaded chromHMM 18-state data of HMEC breast epithelial cells (E119), HeLaS3 cervix cancer cells (E117), colon tissue (E106), HepG2 cells (E118), A549 lung cancer cells (E114), and gastric tissue (E094) from the ROADMAP Epigenomics Project. We counted the number of DREs overlapping with each chromatin state. For each chromatin state, the enrichment fold change and significance were computed by hypergeometric testing using the total CpG probes on HM450 array as control.

We used the hypergeometric test to calculate the statistical significance of the over-represented chromatin state for the DREs. We assigned N as the total number of probes in the HM450 array and K as the number of probes overlapping with the chromatin state under test, n as the number of DREs from N probes that can regulate its target genes, and x as the number of DREs overlapping with the chromatin state under test. The enrichment fold change was calculated as ratio between x/n and K/N. The over-enrichment of chromatin states in DREs was calculated with hypergeometric distribution.

### Histone modifications, sequence conservation, and DNase I hypersensitivity

In order to systematically benchmark the DREs we identified, we collected epigenomic data of various human cells and tissues from the ENCODE Project (Additional file 9: Table S8). We downloaded chromatin marks including histone modifications of H3K4me1, H3K4me3, H3K9me3, H3K27me3, H3K27ac, and p300 ChIP-seq signals to evaluate the enhancer activity of distal DREs, from breast cancer cells (MCF-7), colon cancer cells (HCT116), cervical cancer cells (HeLa-S3), liver cancer cells (HepG2), and lung cancer cells (A549). The enrichment of histone marks at the distal DREs derived from TCGA cancer cohorts was calculated with the epigenome profiling data from the corresponding cell lines or tissues. To evaluate the status of evolutionary conservation, we obtained the 100-way PhastCons conservation data to calculate the conservation score for the distal DREs in each cancer. We have also tested DNase I hypersensitivity data from MCF7, HelaS3, A549, and HepG2. We calculated the scores for each genomic feature on genomic regions 6000 bp flanking each DRE, then got the average score for all DREs from the same cancer cell line.

### Precision of DRE-target pairs

We computed the precision of DRE-target pair predictions by comparing them to the enhancer-promoter pairs (EP-pairs) predicted by chromatin interactions derived from IM-PET, ChIA-PET, Hi-C, and RAD21-cohesin. These tools mainly detect active enhancers with enrichment of active histone marks, such as H3K4me1, H3K4me3, and K3K27Ac, which were confirmed to be enriched in our DREs negatively correlated its targets (Fig. 2d). Other studies show that active enhancers with low DNA methylation tend to have gene targets with high expression [35–38]. DREs positively correlated with its targets were enriched for genomic repressive regions and TF repressors (Fig. 2c and Additional file 1: Figures S2 and S14), but not enriched for active histone marks. This suggested that DREs positively correlated its targets may use different mechanism to indirectly regulate gene expression. Herein, we only considered DREs negatively correlated with its targets for further analysis, similar to other studies [9, 10]. A predicted DRE-target pair will be counted as confirmed if its DRE and target gene overlapped the two ends of an interaction from the IM-PET, ChIA-PET, HiC, or RAD21-cohesin data [23]. The precision result was similar but superior to the result obtained through other methods (e.g. ELMER [9]) (Additional file 1: Figure S3b). Of note, our method output many more negatively correlated EP pairs compared with ELMER (Additional file 1: Figure S3). The datasets of IM-PET, ChIA-PET, and HiC were downloaded from the 4DGenome database [39]. A supplement of HiC data was downloaded from GEO (GSE63525) and ChIA-PET data were downloaded from ENCODE (ENCSR436IAJ). We used the similar procedure [23] to conduct the RAD21-cohesin interaction analysis (termed as CNC), which used ChIP-Seq data to find pairs of cohesin binding-sites that do not contain CTCF sites. The ChIP-Seq datasets of CTCF and RAD21 were downloaded from ENCODE (ENCFF095BZW, ENCFF001TTK, ENCFF001UNO, ENCFF059UOO, ENCFF594DJD, ENCFF001XLM, ENCFF001TTJ, ENCFF001TTK, ENCFF001VDS).

### Comparing MICMIC with other methods

We used IM-PET 23,106 EP interaction pairs between 5311 CpG probes and 344 genes as positive control and tested the precision of EP prediction from patient data by four methods: MICMIC; ELMER; BNstruct (Bayesian Network Structure Learning) [40]; and NEO2 (Network Edge Orienting (NEO) Software) [41]. All the methods were applied on the expression and methylation data from the same patient cohort of TCGA liver cancer. The MICMIC EP prediction was ranked by the normalized mutual information and conditional mutual information. The ELMER EP prediction was ranked by the empirical *p* value (Pe). The BNstruct EP prediction was ranked by the confidence threshold (alpha). The NEO2 EP prediction was ranked by edge orienting score (LEO.NB.OCA). The precision rates were calculated and compared when selecting the same number of top ranked EP pairs from different methods.

### Cell culture

Gastric cancer cell line AGS was from ATCC and liver cancer cell lines BEL-7402 and PLC8024 were obtained from the Institute of Virology of the Chinese Academy of Medical Sciences (Beijing, China). AGS cells were cultured in RPMI-1640 medium (Gibco) supplemented with 10% fetal bovine serum (HyClone) and 1% Anti-Anti (Gibco). BEL-7402 and PLC8024 cells were cultured in DMEM medium (Gibco) supplemented with 10% fetal bovine serum and 1% Anti-Anti. The AGS cell line can be effectively transiently transfected with efficiency > 50% with lipofectamine3000. We selected liver cancer cell line BEL-7402 to test the effect of downregulation of tumor suppressors, such as KLF9, APOA1, APOC3, and CBFA2T3. We used liver cancer cell line PLC8024, a more aggressive one compared with BEL-7402, to test the effect of downregulation of oncogenes, such as HDCA11, CDT1, NDRG1, TKT, TK1, BIRC5, and SLC16A3.

### RNA purification and qPCR

Total RNA was purified using the method described previously [42], followed by treatment with RNase-free DNaseI (NEB). RevertAid RT Reverse Transcription Kit (Thermo) was used to perform the first strand cDNA synthesis according to the manufacturer’s instructions. For qPCR analysis, cDNA was subjected to quantification by iTaq Universal SYBR green supermix (Bio-Rad).

### Plasmids and cloning

Catalytic domains of Dnmt3a and Dnmt3l were amplified from mouse cDNA and were fused to form Dnmt3A-3 L. PUFa from pAC1405-pCR8-4xNLS_PUFa_2xNLS (Addgene #71903) were fused with Dnmt3a-3 l into vector of pcDNA3-Flag-HA (Addgene #10792, a gift from William Sellers). gRNAs were cloned into pAC1371-pX-sgRNA-5xPBSa (Addgene #71888, Additional file 4: Table. S3 for sgRNA sequences). pAC1405-pCR8-4xNLS_PUFa_2xNLS and pAC1371-pX-sgRNA-5xPBSa were gifts from Albert Cheng (Addgene plasmid #71888, Addgene #71903). dCas9 expression plasmid was generated by replacement of the cas9 with dCas9 cassette in px330 vector (px330, Addgene plasmid #42230, a gift from Feng Zhang; 3xFLAG-dCas9/pMXs-neo Addgene plasmid #51260, a gift from Hodaka Fujii). We generated catalytically inactive Dnmt3a (P705V and C706D mutations) by point mutagenesis with primers: Dmt3a-muP705-Forward, GGC AGT GTC GAC AAT GAC CTC TCC ATT GTC AAC CCT G; Dmt3a-muP705-Reverse, TCA TTG TCG ACA CTG CCT CCA ATC ACC AGG, with sequencing confirmation.

Putative distal regulatory regions and promoters of the target genes were amplified from human genomic DNA (see Additional file 4: Table S3 for primer sequences used in cloning) and inserted into the pGL3-basic vector (Promega).

For dCas9-TET1 targeting, we used these plasmids: pCAG-dCas9-5xPlat2AflD and pCAG-scFvGCN4sfGFPTET1CD (Addgene plasmid #82560 and #82561, gifts from Izuho Hatada). We generated catalytically inactive TET1 with H1671Y and D1673A mutations with primers:

Tet1-muH1671-Forward, TCC CTA CAG GGC CAT TCA CAA CAT GAA TAA TGG AAG CAC TG; and Tet1-muH1671-Reverse, AAT GGC CCT GTA GGG ATG AGC ACA GAA GTC CAG, with sequencing confirmation.

Before we decided to use single sgRNA to target one distal DRE, we tested two or three sgRNAs in combination to target one distal DRE. However, there is no difference for the dCas9 targeting effect on the target gene expression.

### Transfections and control design

All transfections were done with lipofectamine 3000 (Invitrogen) according to the manufacturer’s instructions. The ratios of co-transfected plasmids were as follows: 1 gRNAs: 2 px330-dCas9: 1 pcDNA3-Dnmt3A-3 L (test) or pcDNA3 (control) for qPCR; 1 gRNAs: 1 pCAG-dCas9-5xPlat2AflD: 1 pCAG-scFvGCN4sfGFPTET1CD (test) or pcDNA3 (control) for qPCR; 19 pGL3-promoter or pGL3-promoter-enhancer: 1 pRL-TK for luciferase assay; and 5 gRNAs: 10 Px330-dCas9: 5 pcDNA3-Dnmt3A-3 L (test) or pcDNA3 (control): 19 pGL3-promoter or pGL3-promoter-enhancer: 1 pRL-TK for luciferase assay.

Above “pcDNA3 (control)” is a control for dCas9 targeting, in which dCas9 co-transfected with empty pcDNA3 without DNMT3A-3 L/TET1. The same conclusion as shown in Figs. 3d and 4c can be reached by using scrambled sgRNA as the control for the qPCR test (Additional file 1: Figure S8c). dCas9 targeting specificity was confirmed with off-target test by bisulfite sequencing of non-targeted sites (WNT5B-sgRNA in Additional file 1: Figure S7a vs Fig. 3c, and NDRG1-sgRNA in Additional file 1: Figure S7b). dCas9 targeting specificity was also confirmed with qPCR quantifying other non-targeted genes with WNT5B-sgRNA (Additional file 1: Figure S7c). Furthermore, we performed experiments by using “untargeted” or catalytically inactive DNMT3A-3 L/TET1 to rule out the possibility of off-target due to overexpression DNMT3A-3 L/TET1 (Additional file 1: Figure S8a,b). The “untargeted” constructs were generated by removal of the PUFa linker from DNMT3A-3 L-fusion, or removal of scFv linker from TET1-fusion (Additional file 1: Figure S6). For these “untargeted,” catalytically active DNMT3A-3 L/TET1 was overexpressed but targeted to nowhere due to the deletion of “linker” domain. The “untargeted” or catalytically inactive DNMT3A-3 L/TET1 did not result in any significant change of the target gene expression (Additional file 1: Figure S8a, b).

### cDNA cloning and overexpression in lentivirus

We cloned HDAC11, WNT5B, and MLEC cDNA from human cDNA library. We then inserted each cDNA into lentiviral expression vector lenti-Blast, modified from lentiCas9-Blast (Addgene #52962, a gift from Feng Zhang). The lentivirus was packed with plasmids pMD2.G and psPAX2 after co-transfection into 293 T cells. All cDNAs have been confirmed by DNA sequencing.

### Dual luciferase assay

The Dual-Luciferase Reporter Assay System (Promega) was used in dual luciferase assay according to the manufacturer’s instructions.

### Migration assay

4 × 10^5^ of AGS cells or 1 × 10^6^ BEL-7402 and PLC8024 cells were used to conduct migration assay using the 12-Well Chemotaxis Chamber (Neuro Probe) according to the manufacturer’s instructions.

### Cell proliferation assay

CCk-8 (Dojindo) was used to perform cell proliferation assay following the manufacturer’s instructions.

### 5-aza-deoxycytidine treatment

AGS, BEL-7402, and PLC8024 cell lines were treated with 10 μM 5-aza-dC (Sigma-Aldrich) for 48 h, followed by RNA purification and qRT-PCR as described. DMSO was used as a control to establish baseline expression.

### Identification of enriched transcription factor bindings

For a distal DRE-target pair, a TF is considered a regulator of the target if the cognate binding motif of this TF can be found on the ± 250-bp genomic regions flanking the DRE. To identify TFs associated with the ± 250-bp genomic region flanking each DRE, we used TFs from the Mocap database, containing genomic mapping for 823 TFs [43] with binding quality. Stringent cut-off (*p* value < 1e-5) was applied to select the TF binding sites. Mocap method is an integrated classifier that assembles motif scores, chromatin accessibility, TF footprints, evolutionary conservation, and other factors to predict TF bindings. For each DRE category tested (negative-up, negative-down, positive-up, or positive-down), we counted the number of DREs containing the binding site of the TF being tested, denoted as variable “a” below and variable “b” for number of DREs not containing the TF being tested. For the entire DREs combining the four subgroups, we can also get similar number as “c” and “d” for containing and not containing the TF being tested, respectively. Calculation of the enrichment odds ratio (OR) and a 95% confidence interval (CI) was conducted with the following formulas:

$$ OR=\left(a/c\right)/\left(b/d\right) $$

$$ CI=\exp \left(\log (OR)\pm 1.96\sqrt{1/a+1/b+1/c+1/d}\right) $$

We then filtered TFs with an OR > 1.05 as the enriched TFs in each DRE category.

### Evaluate the tissue specificity of genes

We downloaded the gene expression data of human tissues from GTEx (GTEx V6 dataset) [44]. We used the Voom method to normalize the data and limma [31] to identify the differential expression genes comparing samples of one tissue against all other tissues. Genes passing the threshold, log2 transformed Fold-Chang > 0.58 or < − 0.58 and adjusted *p* value < 0.01, were identified as the tissue specific ones.

### Enrichment of transcription repressors

We searched the AmiGO database [45] with the key words “transcription repressor” and “negative regulation” to obtain a list of genes related to the transcriptional repression process and collected the repressor information from GO:0017053, GO:0090571, GO:0001206, GO:0001227, GO:0001191, GO:0000900, GO:0070491, GO:0070176, GO:0003714,GO:0032785, GO:2000143, GO:1903507, and GO:0001078. These gene sets include transcriptional repressor activity, translation repressor activity, and transcription repressor complex. Enrichment of transcription repressor of TFs associated with distal DREs was conducted by hypergeometric analysis.

### Discovery of core transcriptional regulatory circuitry

Core regulatory circuitry (CRCs) is formed by a set of key TFs associated with super-enhancers (SEs) in regulating tumor-subtype specific gene expression and maintaining tumor cellular identity. The TFs in each CRC are auto-regulated by themselves via binding sites on their corresponding SE. The TFs can also cross-regulate each other by forming an interconnected loop via cognate binding sites on other TFs’ related SEs. Based on this information, we took advantage of the genome-wide information on distal DRE-targets generated from our MICMIC method to assemble the CRCs regulated by DNA methylation for each cancer type. The information for SEs of human genome hg19 was downloaded from dbSUPER [46]. First, we selected the distal DREs overlapping with SEs and identified the enriched TFs (OR > 1.05, CI = 95%) associated with these distal DREs. We then predicted the auto-regulatory loops with the following criterion: the TF on test is under regulation of distal DREs with binding sites for TF itself. Cross-regulation between a pair of TFs can be inferred if the cognate binding site of one TF can be found on the other TF’s related SE. After putting together all of the auto- and cross-regulations, we generated an interconnected CRCs eventually.

### TF targets and downstream cancer pathway analysis

As mentioned before, for a distal DRE-target pair, a TF is considered a regulator of the target if the cognate binding motif of this TF can be found on the surrounding regions of the DRE (± 250 bp). After identification of the targets for CRCs, we conducted enrichment analysis for the downstream pathways. Enrichment analysis of KEGG cancer pathways was conducted to identify the pathway targeted by CRC TFs highly expressed in each tumor subtype (cut-off *p* value < 0.05).

### TF network decomposition and network motif identification

The TF network mediated by distal DREs was derived from genome-wide DRE-target information predicted by MICMIC after removal of non-TF genes. For network motif analysis, we used the mfinder software [47] to disassemble the TF network. On average across the 11 cancer types, the TF networks were decomposed into 1.85 million three-node subgraphs with 13 types of three-node network motifs identified. Relative enrichment or depletion of each of the 13 basic network motifs within each cancer was calculated. Two hundred randomized same-size networks were used as the random control and the significance Z-score was calculated (Z > 2 considered as enriched and Z < − 2 as depleted).

## Additional files

Additional file 1: Supplementary figures. (DOCX 10287 kb)

Additional file 2: **Table S1.** Barcode of TCGA samples. (XLSX 81 kb)

Additional file 3: **Table S2.** DREs identified from 11 cancers. (XLSX 5825 kb)

Additional file 4: **Table S3.** sgRNA-primer of DRE tested. (XLSX 11 kb)

Additional file 5: **Table S4.** Category of methylation change. (XLSX 5313 kb)

Additional file 6: **Table S5.** Interconnected loops in core regulatory circuits. (XLSX 40 kb)

Additional file 7: **Table S6.** TF_network_motifs. (XLSX 11 kb)

Additional file 8: **Table S7.** Cosmic non-coding variations neighboring LIHC DRE. (XLSX 187 kb)

Additional file 9: **Table S8.** Data source of histone marks and DNase I. (XLSX 10 kb)

## Abbreviations

DREs: Direct regulatory elements
MRA: Master regulator analysis
TCGA: The Cancer Genome Atlas
TF: Transcription factor
TSS: Transcription start sites
