# Supplementary material for: MICMIC: identification of DNA methylation of distal regulatory regions with causal effects on tumorigenesis
Source: Genome Biol. 2018 Jun 5;19:73. doi: 10.1186/s13059-018-1442-0 (PMC5989391; doi:10.1186/s13059-018-1442-0)
Supplement: Supplementary file 1 — Supplementary figures. (DOCX 10287 kb) [file 13059_2018_1442_MOESM1_ESM.docx]

**
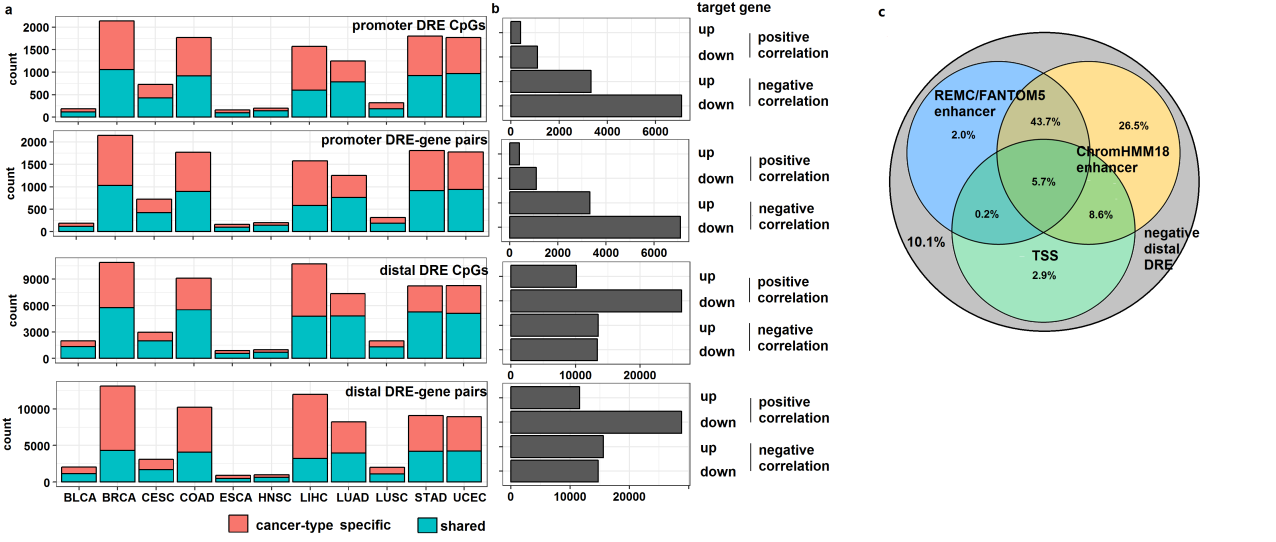
**

**Fig. S1. Summary of cancer-type specific and shared DREs, and DRE-target pairs.**(a) (b)The bar chart showing the number of DRE-target pairs and DREs, promoter or distal, identified from each cancer type. The green color bars indicate the fraction of the DREs or DRE-target pairs shared by one or more cancer types, and the red color bars indicate the fraction of the cancer-type specific DREs or DRE-target pairs. In the right panel, DRE-target pairs are categorized into 4 groups based on the correlation direction (positive or negative) and the expression of target (up or down) in tumor- versus normal-samples. There are more unique DRE-target pairs than the unique DREs since one DRE can regulate multiple gene targets.(c) The distribution of negatively-correlated distal DREs in well-annotated enhancers (REMC enhancer, FANTOM5 enhancers, chromHMM18 genic/active enhancers), and TSS-proximal regions (<2kb to any TSSs, including non-coding RNA/alternative transcriptional starting sites).

**
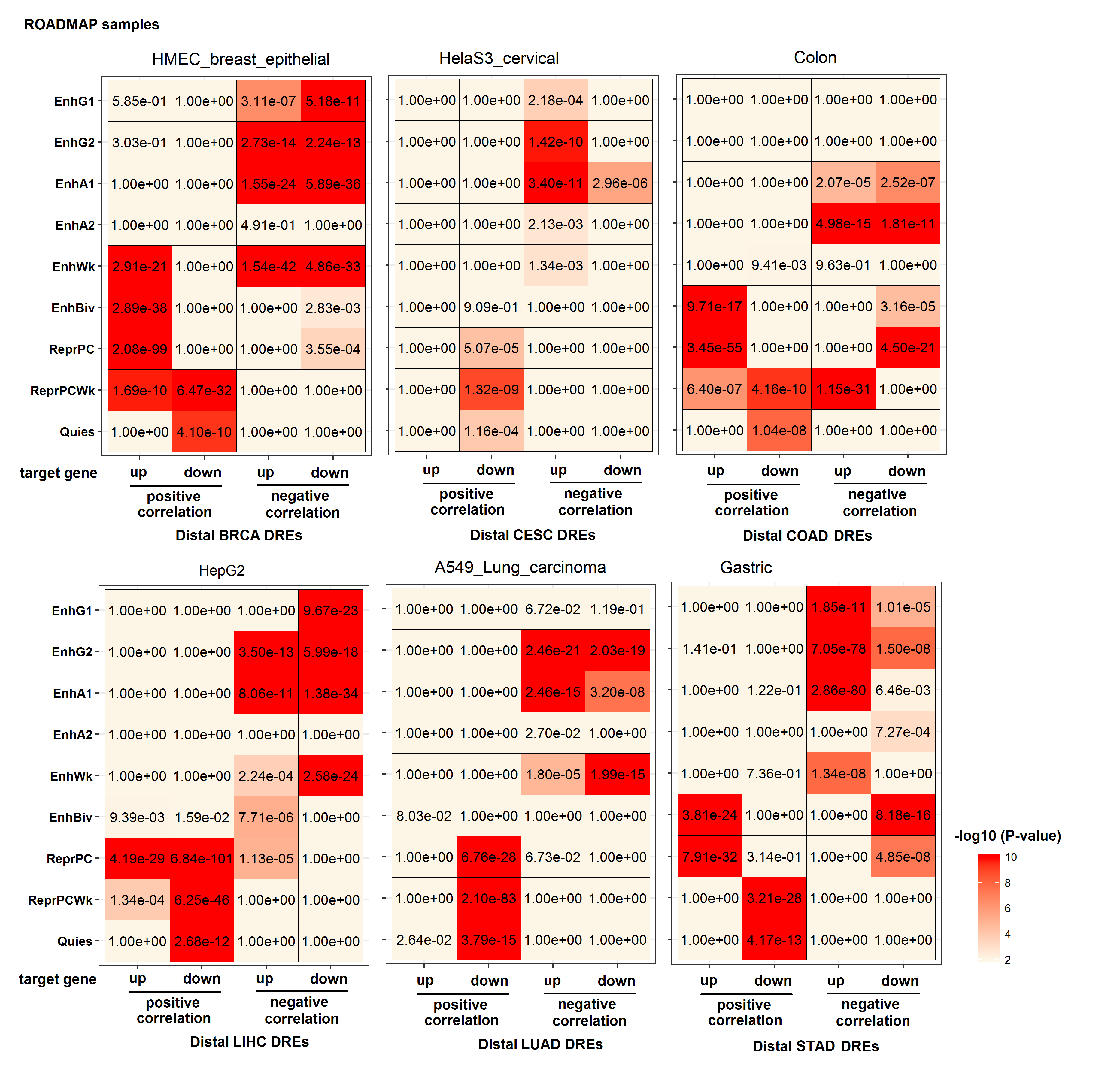
**

**Fig. S2. Chromatin state of distal DREs.** Results showing the preferred chromatin state of distal DREs in the corresponding cancer cell lines. The distal DREs for each cell line were inferred from those identified from the corresponding TCGA cohort. The number of distal DREs were counted at each chromatin state, with the heatmap indicating the enrichment p-value of distal DREs in each state.

**
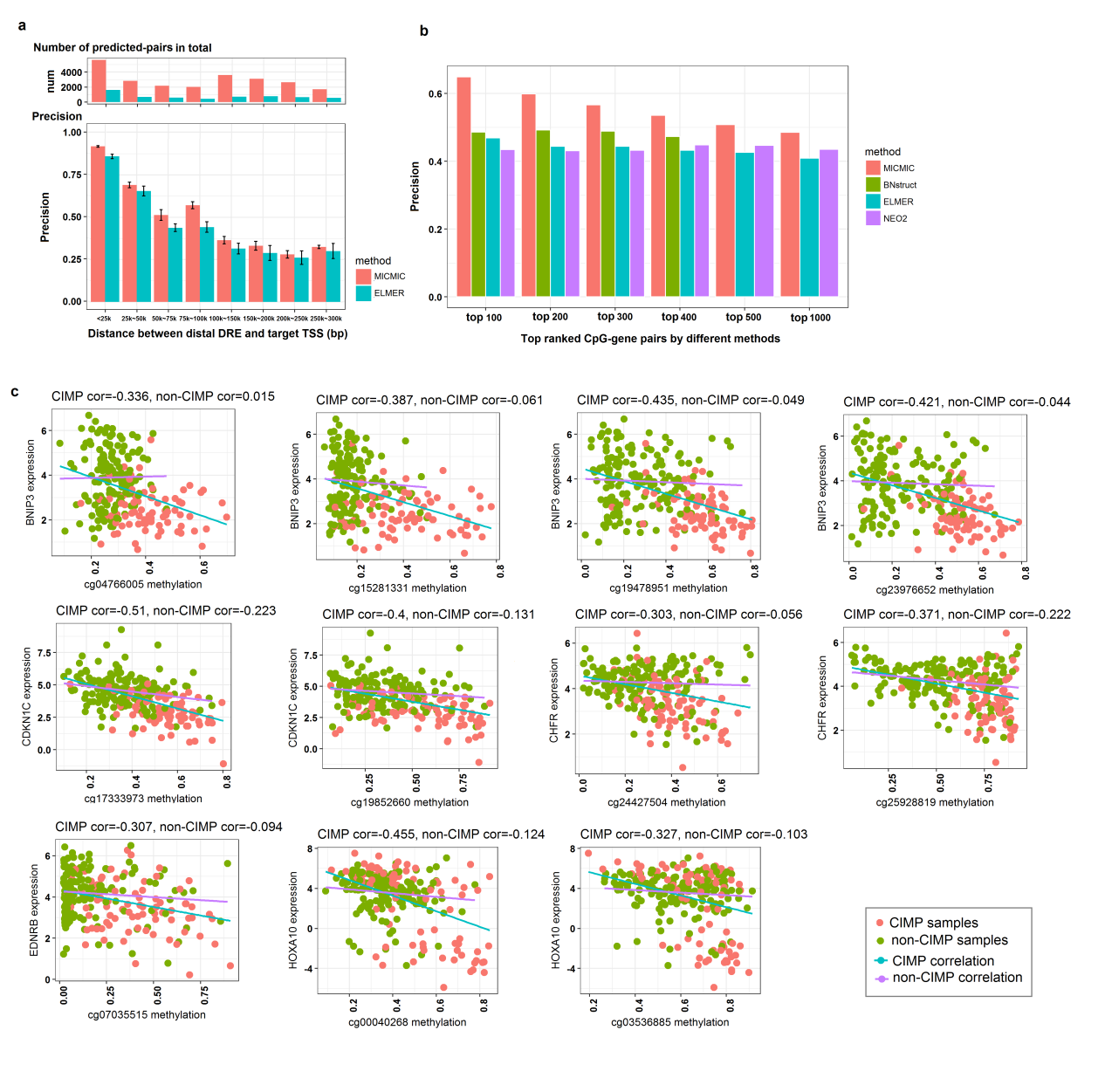
**

**Fig. S3. Performance comparison between MICMIC, ELMER, BNstruct and NEO2, and negative cases identified by MICMIC.** (a) Negatively-correlated DRE-target pairs from MICMIC was compared with the EP pairs (enhancer-promoter, negatively-correlated) identified by ELMER. MICMIC outperformed ELMER with more negatively-correlated DRE-target pairs (top) and higher precision for these pairs (bottom). Standard deviation was calculated by precision results from different cancers.(b) Precision of top rank EP pairs identified by 4 methods applied on the same liver cancer cohort. The top 100 EP pairs identified by MICMIC reached 65% precision, outperforming all other methods. (c) Examples of highly correlated CpGs rejected by conditional mutual information. The down-regulation of each gene was caused by the CpG island methylator phenotype (CIMP), which induced the genome-wide hypermethylation of each CpG. In contrast, there was no correlation in the non-CIMP samples.

**
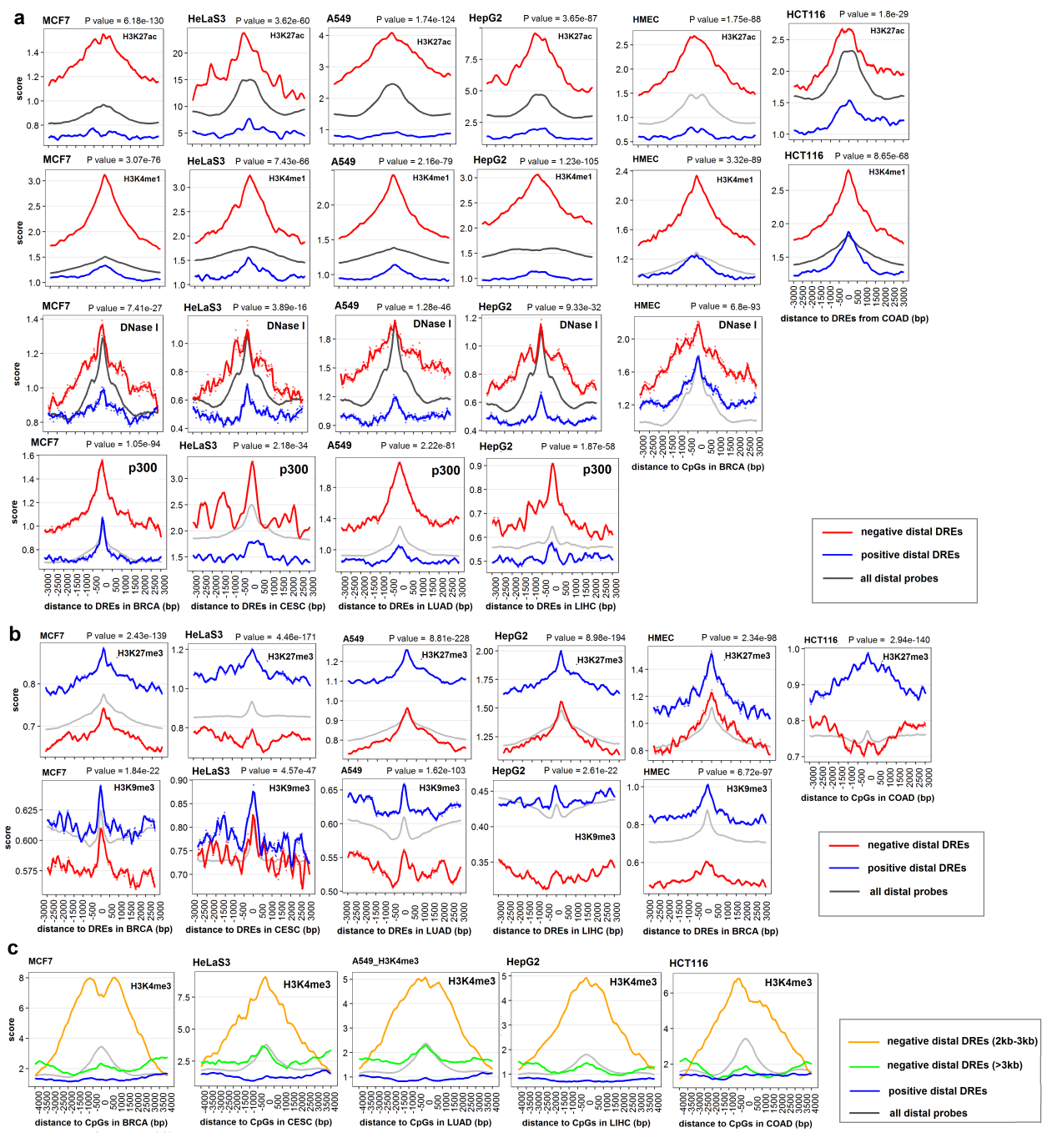
**

**Fig. S4. Enrichment analysis of chromatin marks in genomic regions flanking the distal DREs in the corresponding cancer cell lines**. The distal DREs for each cell line were inferred from those identified from the corresponding TCGA cohort**.** (a) Increased enhancer chromatin signals, such as H3K27ac, H3K4me1, p300 and DNase I hypersensitivity, at genomic regions surrounding the distal DREs negatively correlated with its targets in each cancer type. By contrast, there is little enrichment for DREs positively correlated with its targets. P-values were calculated by t-tests between signals of negative distal DRE versus that of an all probes control.(b) Increased repressive chromatin signals, such as H3K27me3 and H3K9me3, at genomic regions surrounding the distal DREs positively correlated with its targets in each cancer type. By contrast, there is little enrichment for DREs negatively correlated with its targets. (c)Enrichment of H3K4me3, marker of active promoters, was only observed at a minority (<30%) of negatively-correlated DREs, which were 2kb-3kb away from TSS.

**
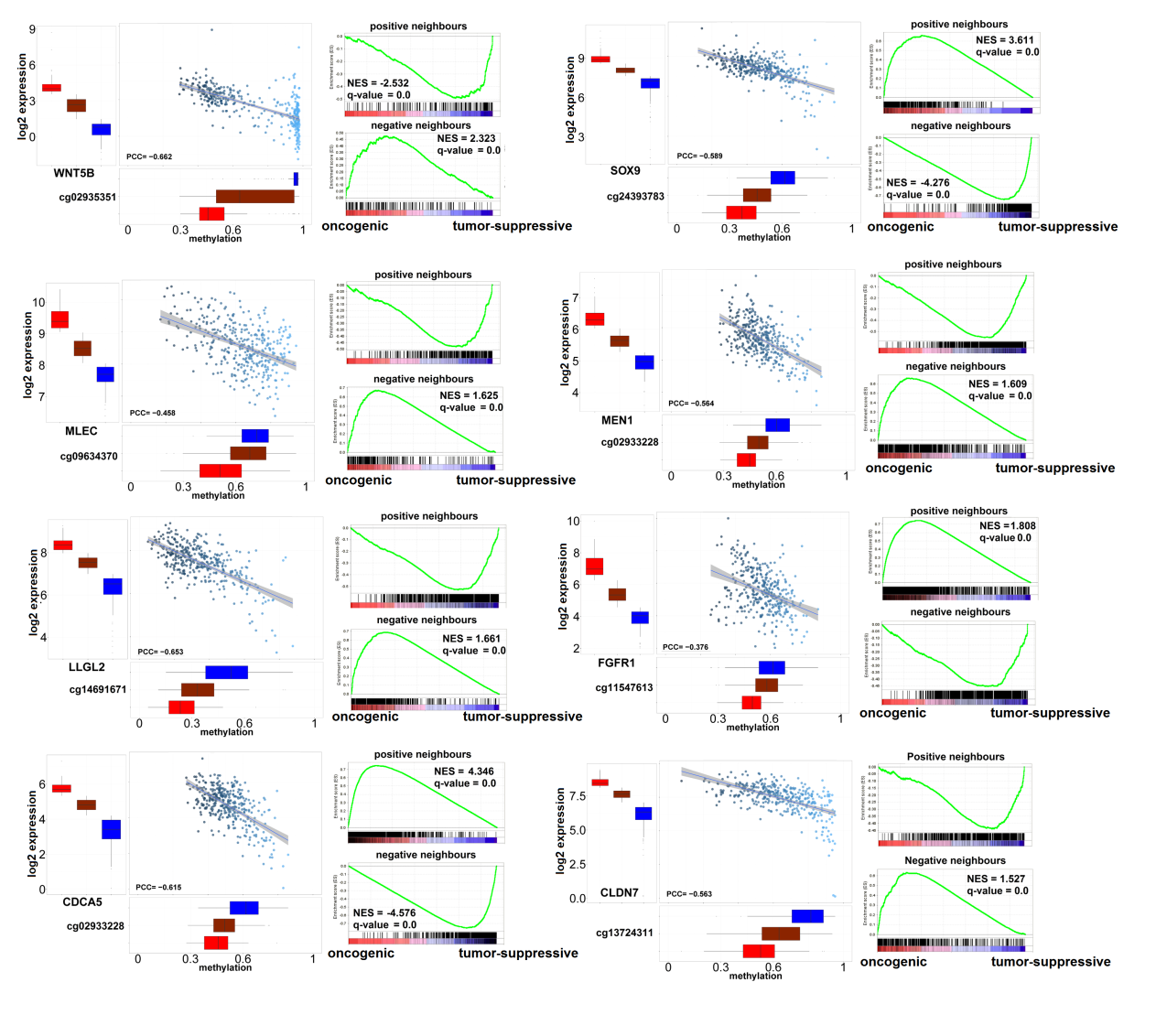
**

**Fig. S5. Expression-methylation correlation of DRE-targets from TCGA STAD cohort and the MRA prediction.** The box- and scatter-plots show the correlation between DRE methylation and its target gene expression in TCGA gastric cancer cohort (STAD). Box plot shows the high, middle and low expression groups of target gene, plotted against the methylation of the distal DRE in each group. GSEA plots by master regulator analysis (MRA) show the enrichment significance of cancer signatures for each gene.

**
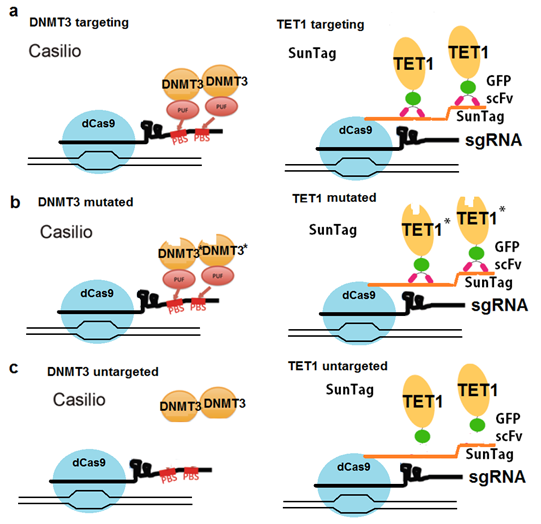
**

**Fig. S6.** Multiple lines of control were included to measure the off-target effect due to overexpression of DNMT3/TET1. The figure above illustrates dCas9-targeting with wild-type TET1/DNMT3 (a), or mutated (catalytically inactive) TET1/DNMT3 (b), or untargeted TET1/DNMT3 by deletion of the “link” domain (PUFa for DNMT3 and scFv for TET1) (c). TET1/DNMT3 in (c) had full enzyme activity, but cannot be targeted to a specific locus due to deletion of the “link” domain. Control (c) was named as “untargeted” here.

**
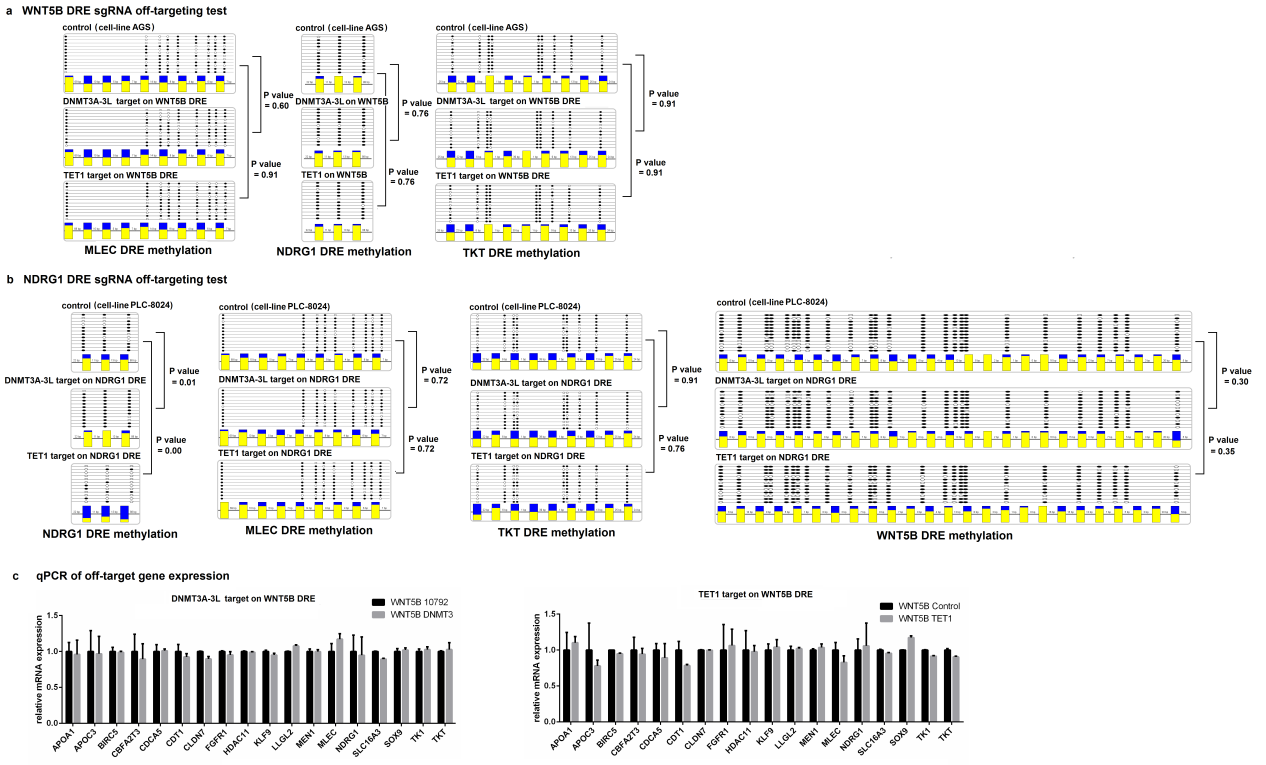
**

**Fig. S7. Bisulfite sequencing and qPCR of off-target tests.** (a) After inducing of dCas9-DNMT3A-3L/TET1 targeting on WNT5B DRE, no significant methylation alteration was detected in the three off-target sites.(b) NDRG1 DRE presented 22% increasing by dCas9-DNMT3A-3L targeting (from 69% to 91% p-value = 0.01), and 44% methylation decreasing by dCas9-TET1 targeting (from 69% to 22%, p-value = 0.00). No significant methylation change was detected around the three off-target sites. (c) For 18 off-target genes, no significant expression change can be observed by WNT5B targeting.

**
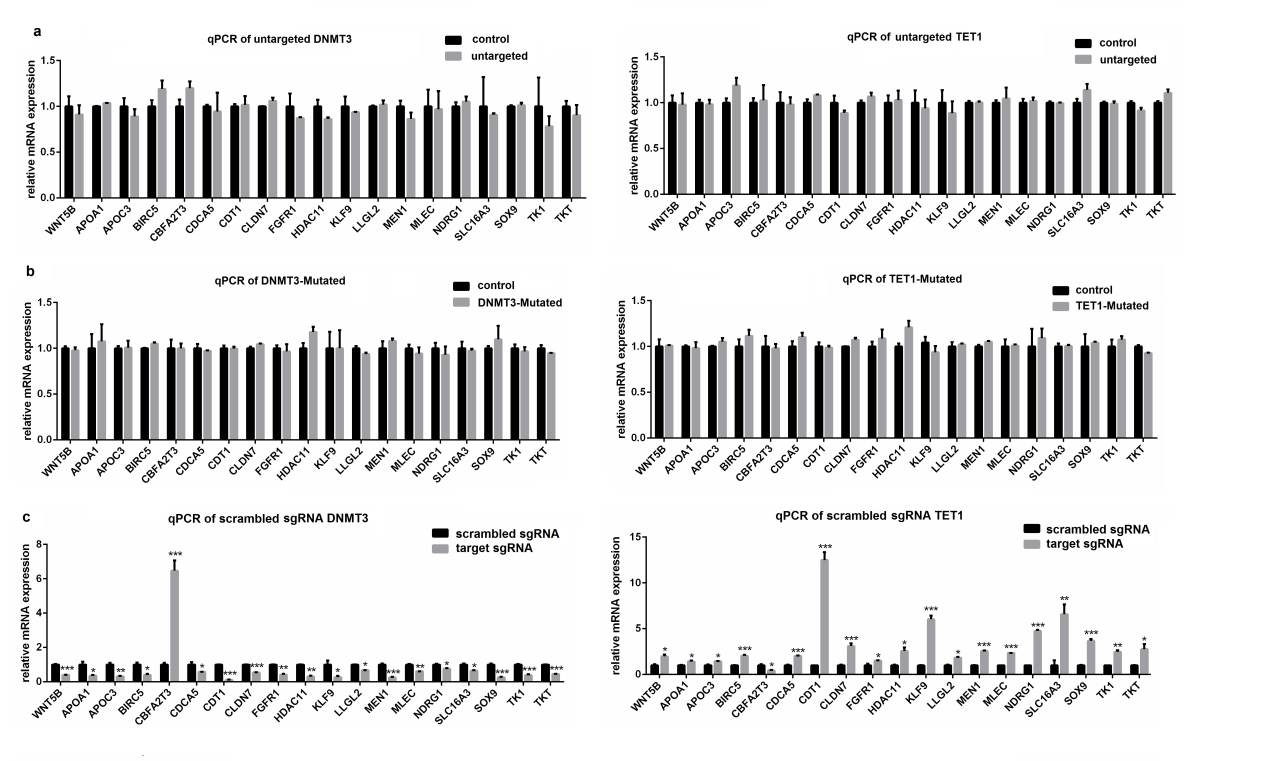
**

**Fig. S8. qPCR quantification of target gene expression in untargeted, mutated and scrambled-sgRNA tests.** (a) Untargeted tests performed by DNMT3A-3L-(no-PUFa) or TET1-(no-scFv),which has intact enzyme activity but unable to be targeted. (b) dCas9-targeting tests with catalytically inactive DNMT3A-3L or TET1 generated by point mutagenesis. (c) qPCR test after DNMT3A-3L/TET1 targeting with scrambled sgRNA as control. Similar results were observed when comparing (c) with Fig. 3d and 4c. “control” in (a) and (b) is dCas9–only (without DNMT3A-3L or TET1), same as “ctr1” or “ctr2” in Fig. 3d or 4c, respectively.

**
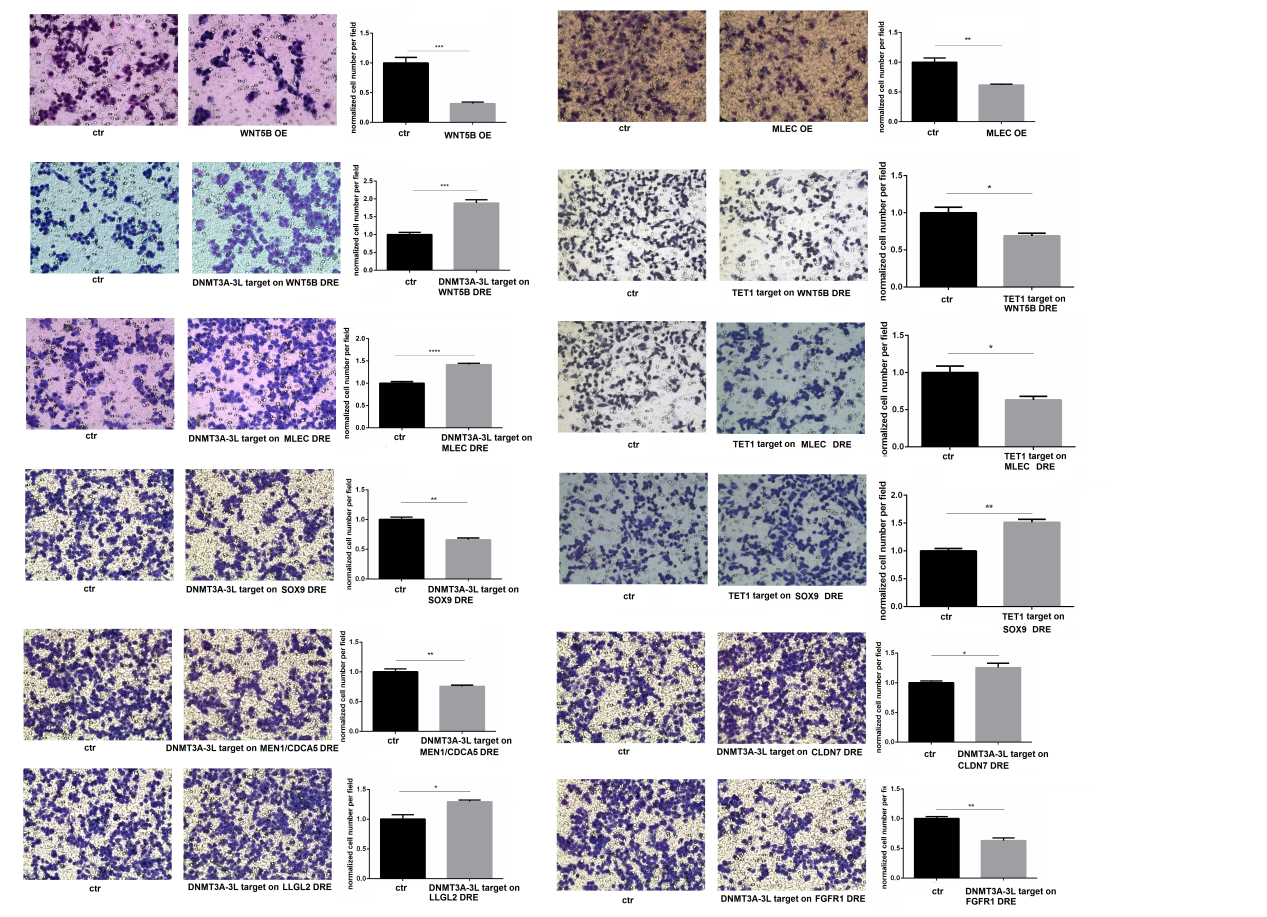
**

**Fig. S9. Cell migration before and after DNMT3A-3L/TET1 targeting to the distal DREs of TCGA STAD gastric cancer.** Cell migration results confirmed the causal effect of DNA methylation of distal DREs on cancer cell malignancy in gastric cancer cells. Distal DRE of each gene was targeted by dCas9-DNMT3A-3L/TET1, which led to the alteration of cell migration. Lentiviral based cDNA overexpression (OE) was performed for WNT5B and MLEC. Cancer cell migration decreased by overexpression of WNT5B or MLEC cDNA, or by TET1 targeting, but increased as a result of DNMT3A-3L targeting.

**
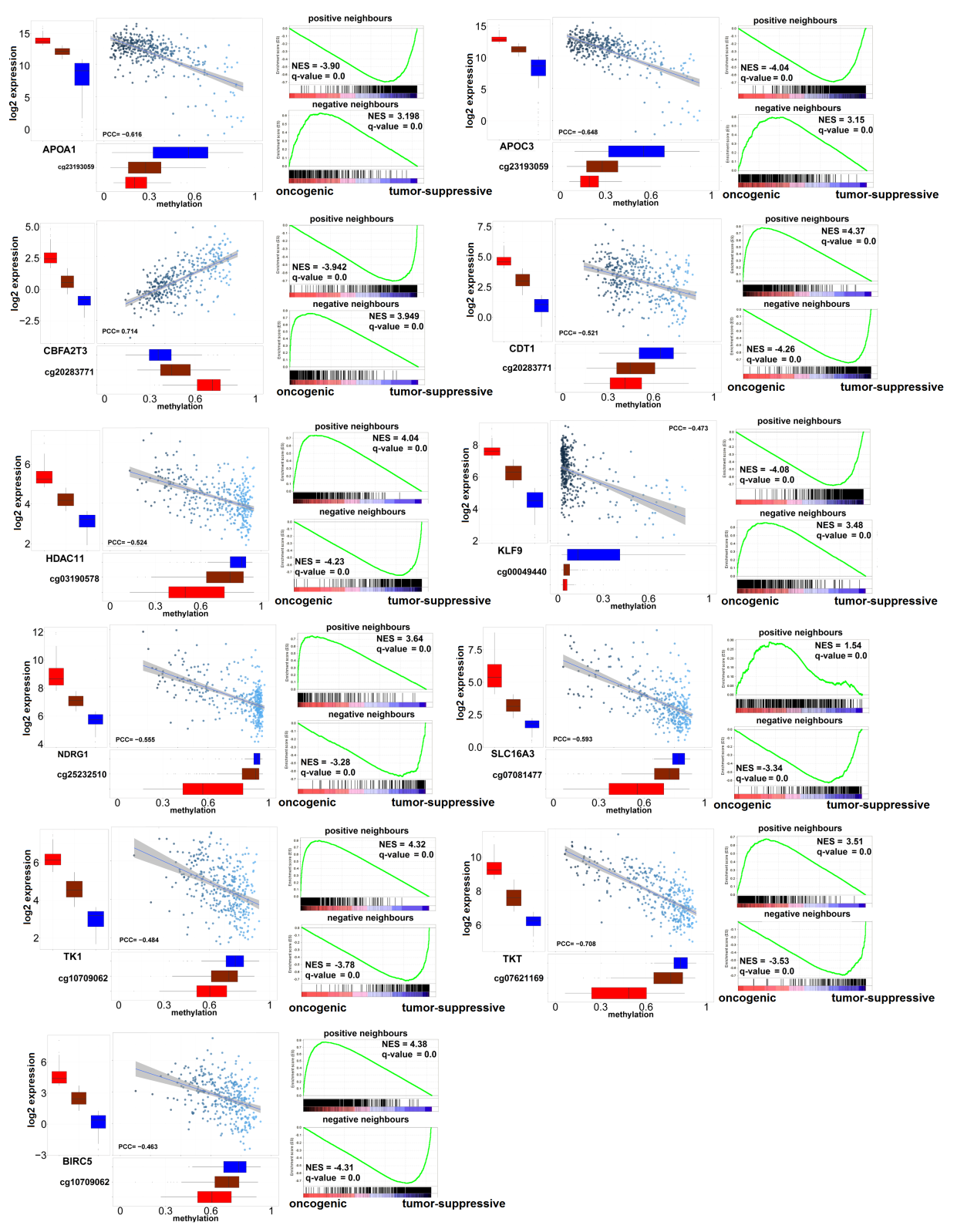
**

**Fig. S10. Expression-methylation correlation of liver cancer (TCGA LIHC) DREs and MRA prediction.** The box- and scatter-plot show the correlation between DRE methylation and target gene expression in TCGA liver cancer cohort LIHC. Box plot shows the high, middle and low expression groups of target gene, plotted against the methylation of the distal DRE in each group. GSEA plots by master regulator analysis show the enrichment significance of cancer signatures for each gene.


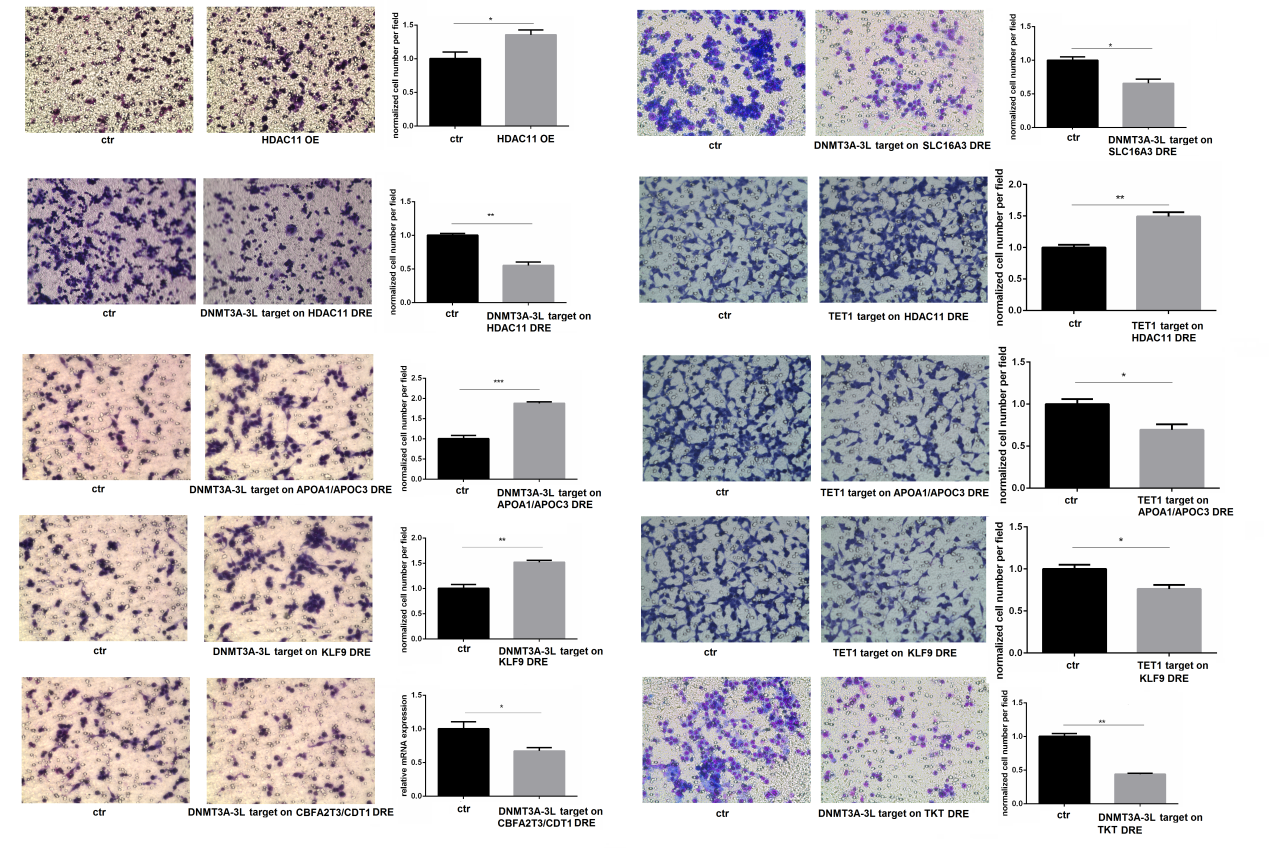


**Fig. S11. Cell migration before and after DNMT3A-3L/TET1 targeting on TCGA liver cancer (LIHC) DREs.** Cell migration results confirm the causal effects of distal DRE methylation on cancer cell malignancy in liver cancer cells. Distal DRE of each gene was targeted by dCas9-DNMT3A-3L/TET1, which led to the alteration of cell migration. Lentiviral based cDNA overexpression (OE) was performed for HDAC11. Cancer cell migration increased by overexpression of HDAC11 cDNA, or by TET1 targeting, but decreased as a result of DNMT3A-3L targeting.

**
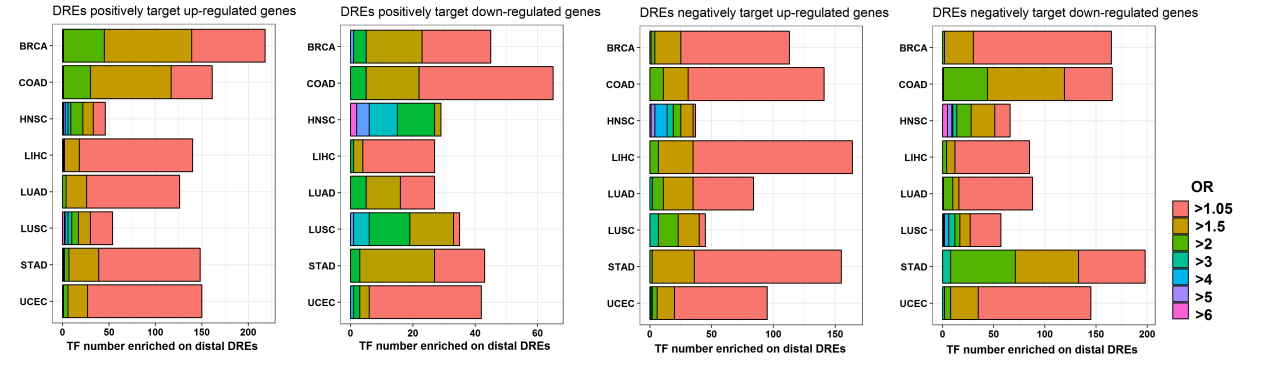
**

**Fig. S12. Number of transcription factors binding on distal DREs.** Numbers of transcription factors enriched in the different categories of distal DREs across cancers. The enrichment odds ratios (ORs) of TFs are presented in colors.

**
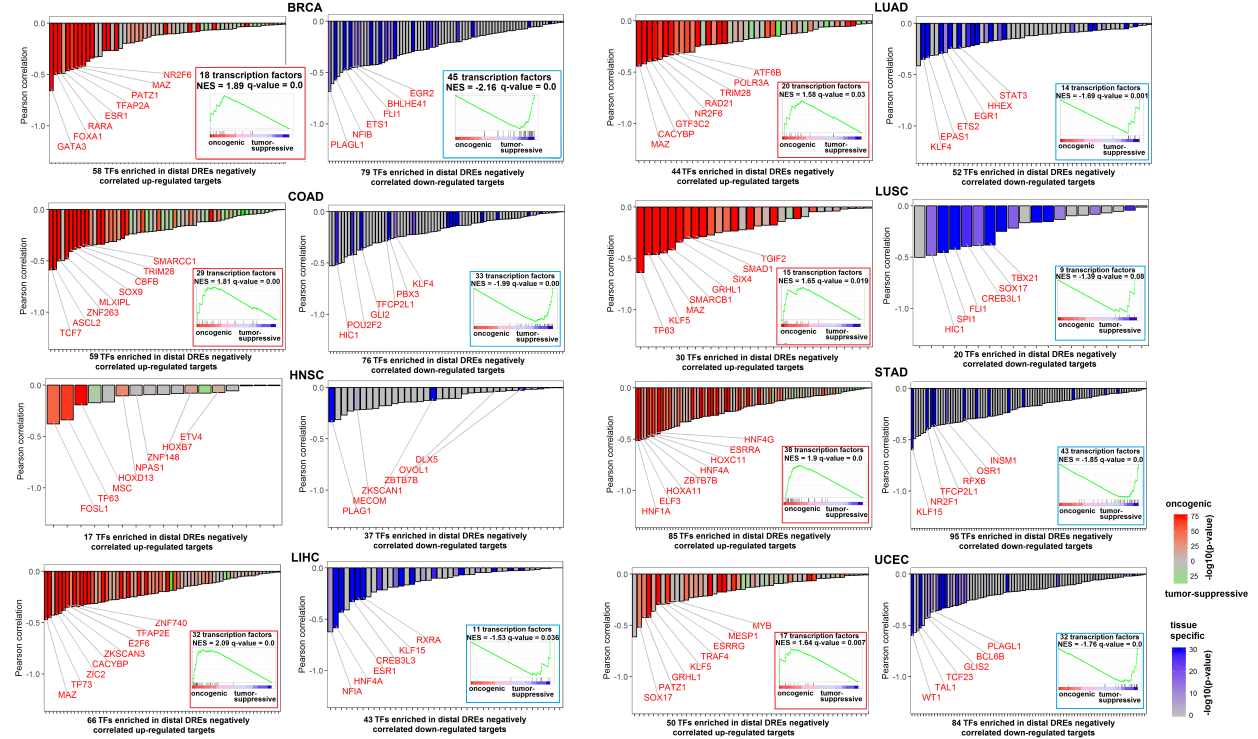
**

**Fig. S13. Distal DRE methylation and the cognate transcription factor binding.** Bar charts show the enriched transcription factors (TFs) that bind to DREs showing a negative correlation between the average methylation of TF binding motifs and TF expression, with down-regulation (negative-down DREs, blue/grey) or up-regulation (negative-up DREs, red/green) of the target gene. Intensity of blue color indicates the degree of tissue specificity of the TF in each cancer type compared to other tissue types. Intensity of red/green color indicates the degree of oncogenic/tumor suppressive behavior of the TF. Bar chart showing the enriched TFs binding on the group-specific distal DREs. The TFs were ranked by the negative correlation between the TF expression and average DNA methylation of the TF binding motifs on the group-specific distal DREs. The correlation value is shown on the y axis. Colors represent the tissue type significance or master regulator significance of the TF gene. The cancer signature association of the top rank TFs (PCC>0.2) is shown in the inset GSEA plot.

**
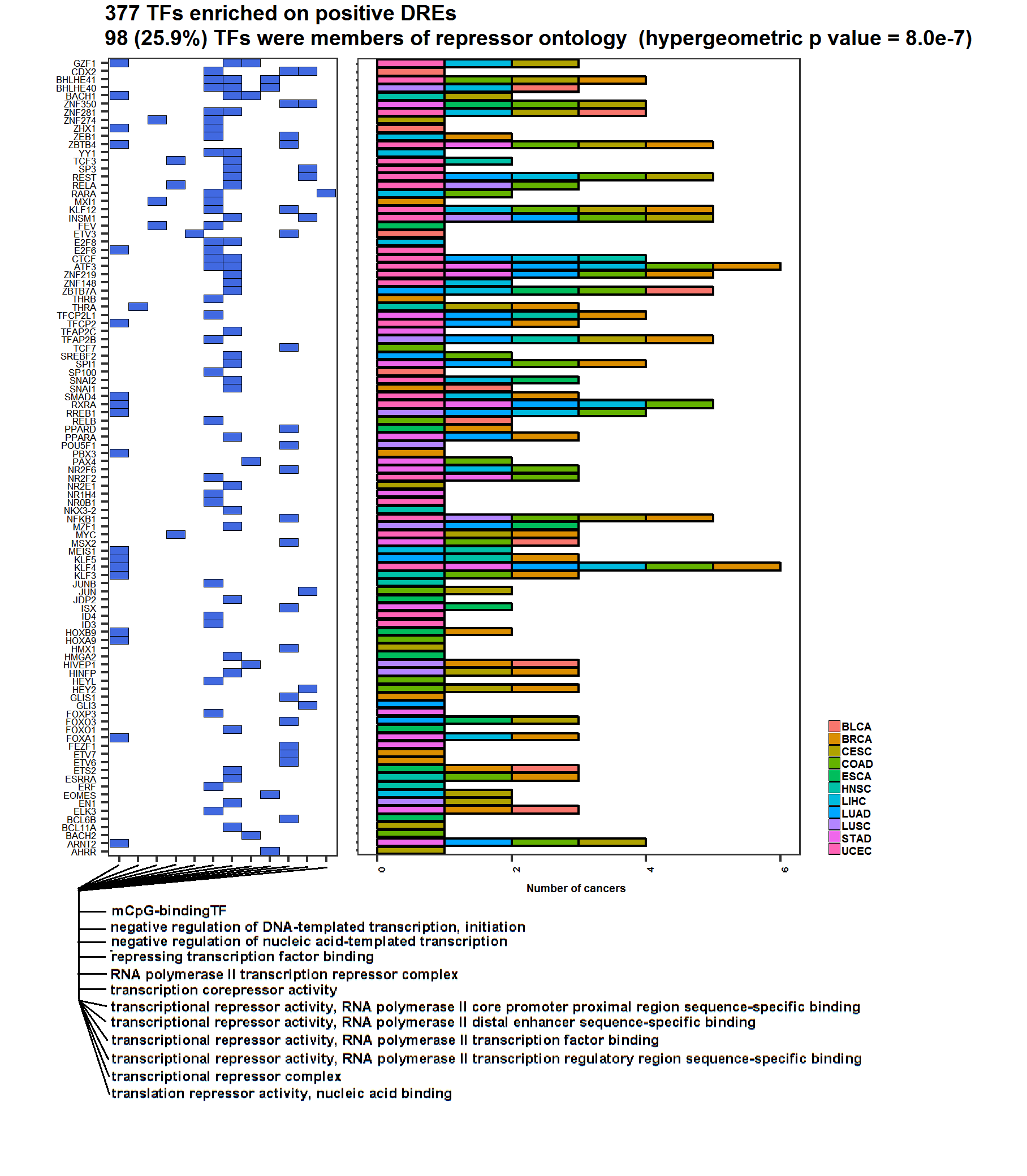
**

**Fig. S14. Transcription repressors enriched in positively-correlated DREs.** Among the total 377 TFs enriched in positively-correlated distal DREs across various cancer types, there are 25.9% (98, p-value = 8e-7) TFs annotated with repressor gene ontology (Online Methods), such as repressors ATF3, REST, RXRA, NFKB1 identified from at least 5 cancer types. This result suggests a novel mechanism that DNA methylation may affect the binding of TF repressors with implications in tumorigenesis. Cancer number is shown in the right panel. Heatmap marks the TFs annotated with repressor ontology. Repressor enrichment p-value is calculated by hypergeometric test using all TFs as control.

**
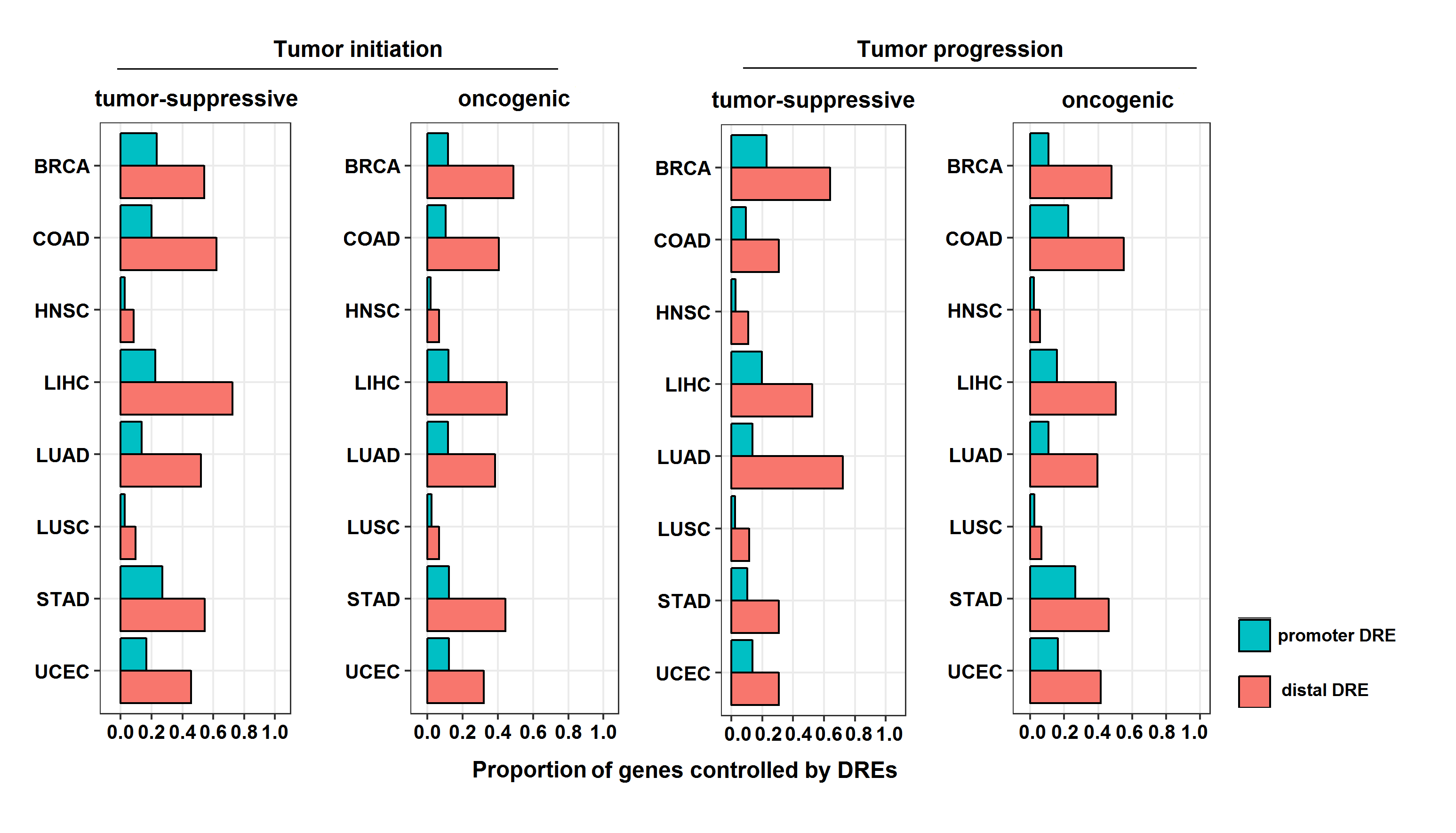
**

**Fig. S15. Proportion of master regulator genes controlled by promoter and distal DREs.** Proportion of tumor-suppressors and oncogenes controlled by promoter and distal DREs. Tumor-suppressors and oncogenes during initiation and progression are identified by master regulator analysis.

**
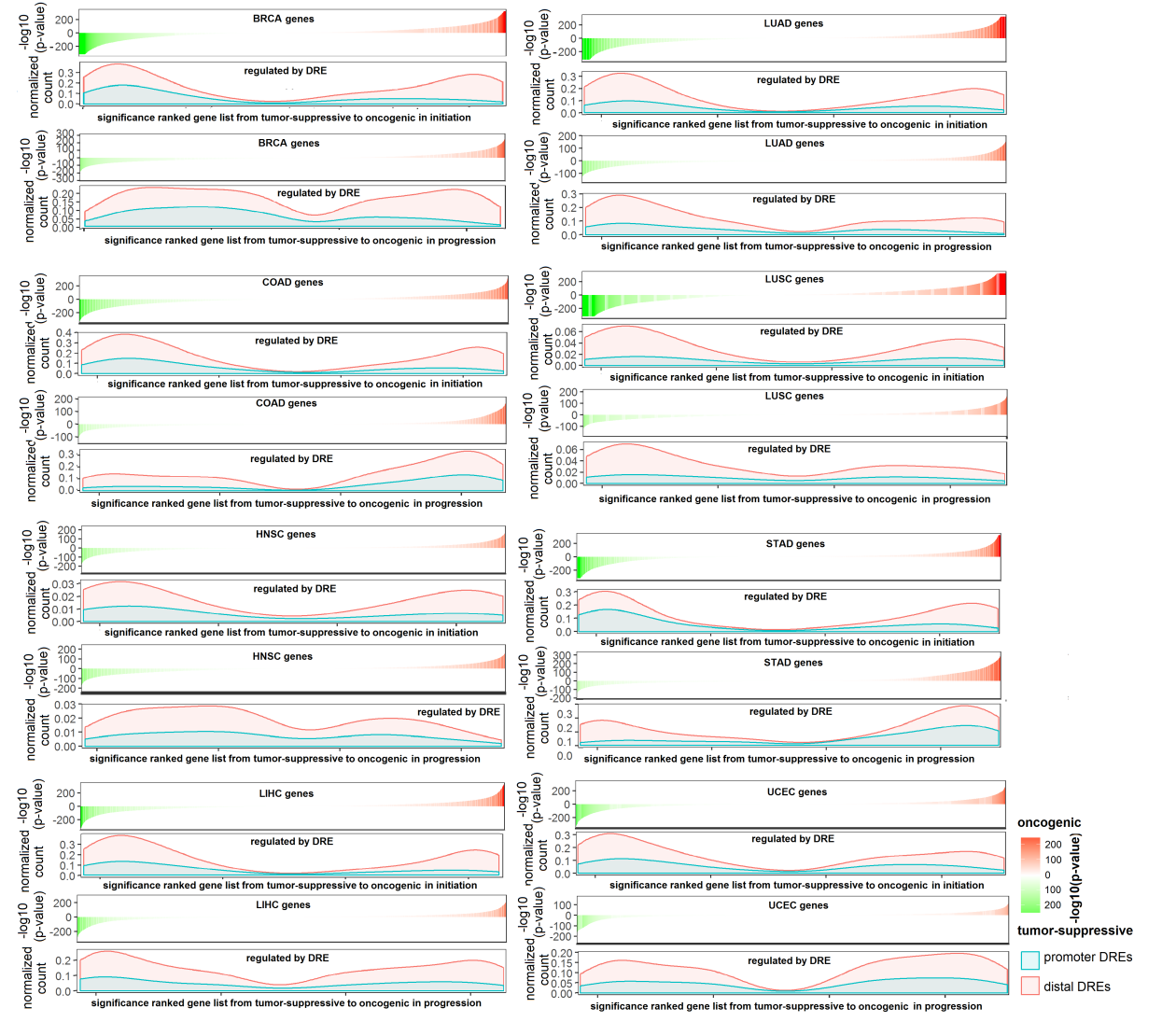
**

**Fig. S16. Higher impact of distal-DREs on cancer genes compared with promoter-DREs during initiation (top) and progression (bottom).** Y-axis of each of the top waterfall panel shows the master regulator significance for each gene, ranked from tumor-suppressive to oncogenic. Y-axis of each bottom panel shows the density normalized gene counts controlled by promoter or distal DREs.

**
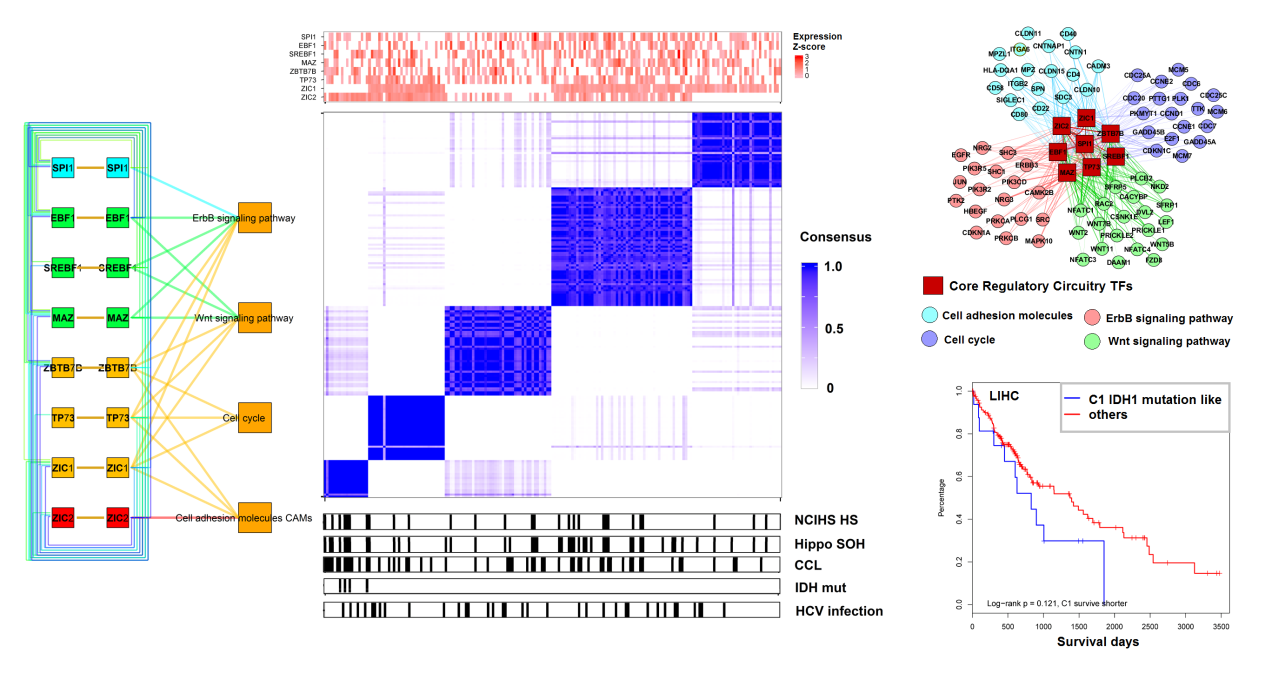
**

**Fig. S17. Tumor-subtype core regulatory circuitry (CRC) in TCGA LIHC liver cancer.** Joint consensus clustering of CRC TFs of LIHC identified the IDH mutation subtype in liver cancer. CRC class 1 exhibited great similarity with the IDH1 mutation type and cholangio carcinoma-like (CCL) subtype, which represent a poorer prognosis subtype of HCC(not significant due to small sample size).

**
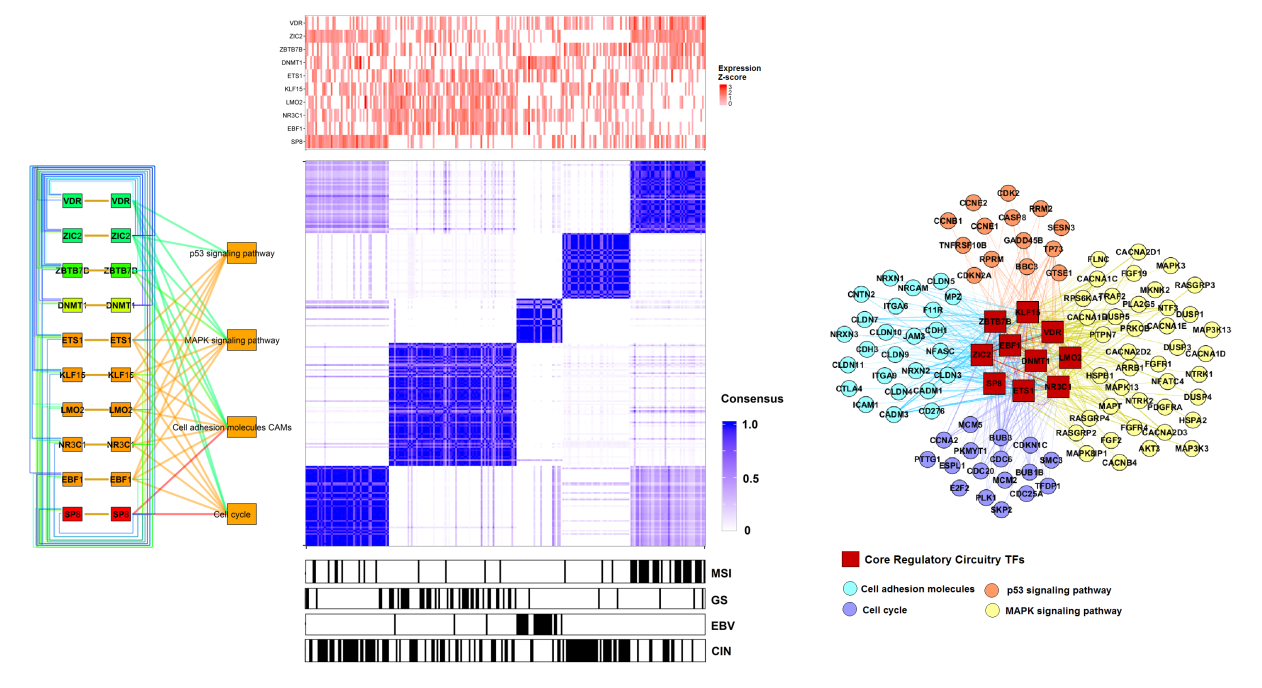
**

**Fig. S18. Tumor-subtype core regulatory circuitry (CRC) in TCGA STAD gastric cancer.** Joint consensus clustering of CRC TFs of STAD recapitulated the four major molecular subtypes: Epstein–Barr virus infection, microsatellite unstable (MSI), genomic stable (GS), chromosomal instability (CIN) of gastric cancer. To identify the underlying signaling pathways regulated by CRC, we further expanded the CRCs network for each cancer type with the TF targets identified from MICMIC. We then applied the enrichment analysis on the targets of the CRC core TFs in different CRC subgroups respectively. In gastric cancer, two TFs of the MSI-like subgroup (VDR and ZIC2) regulated 16 genes in cell adhesion molecules (p value = 0.003), 24 genes in MAPK signaling pathway (p value = 0.01259) and 9 genes in p53 signaling pathways (p value = 0.0127).


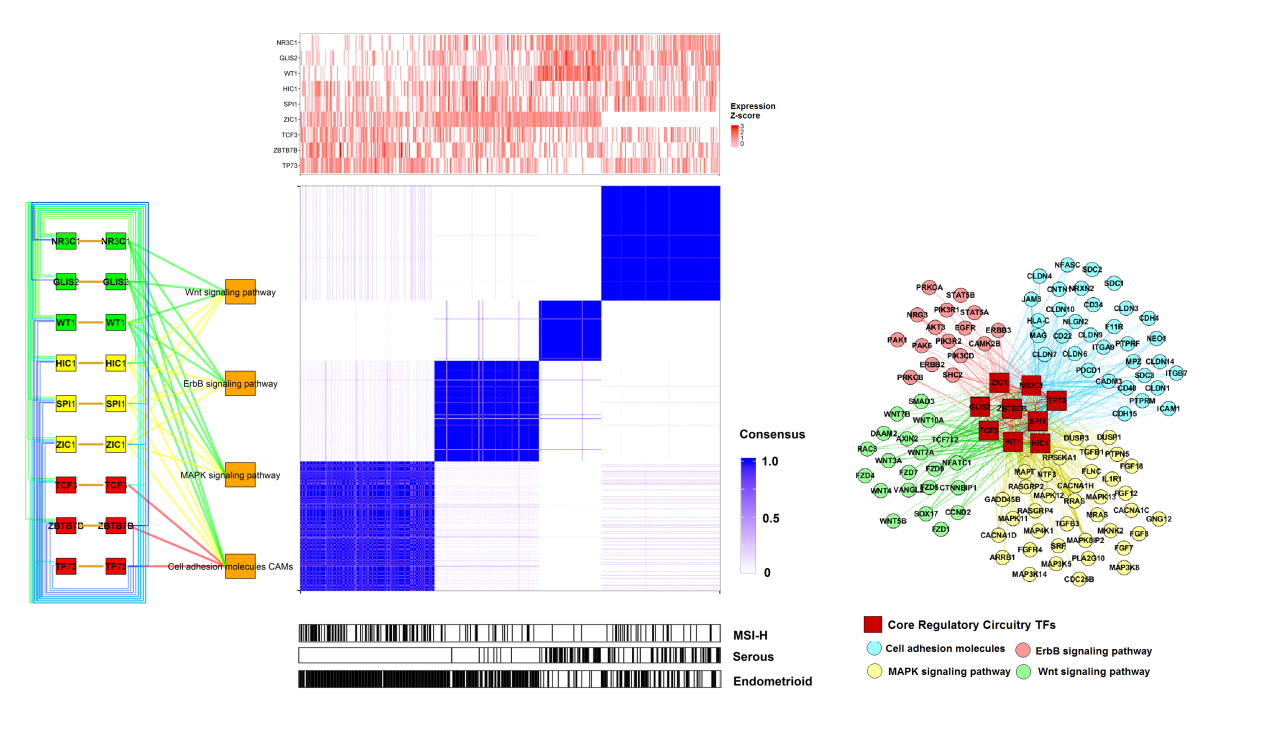


**Fig. S19. Tumor-subtype core regulatory circuitry (CRC) in TCGA UCEC cancer.** Joint consensus clustering of CRC TFs identified four subtypes of TCGA endometrial carcinoma (UCEC), overlapping with known subtypes, such as MSI, serous, and endometrioid. To identify the underlying signaling pathways regulated by CRC, we further expanded the CRCs network for each cancer subtype with the TF targets identified from MICMIC. We then applied the enrichment analysis on the targets of the CRC core TFs in different CRC subgroups respectively. For example, the serous like CRC subgroup was controlled by three TFs (WT1, GLIS2 and NR3C1), targets of which were enriched for cell adhesion molecules (27 targets, p value = 2e-5), WNT signaling pathway (20 targets, p value = 0.03), MAPK signaling pathway (32 targets, p value = 0.03), and ErbB signaling pathway (13 targets, p value = 0.03).
